# Intracellular Aβ42 Sequestration by a Serine Protease Mitigates Neurotoxicity in a *Drosophila* Alzheimer's Disease Model

**DOI:** 10.1002/advs.202517862

**Published:** 2026-03-18

**Authors:** Jingyun Su, Meng Yang, Xinfeng Wang, Pa Wu, Zongzhao Zhai

**Affiliations:** ^1^ Hunan Provincial Key Laboratory of Animal Intestinal Function and Regulation College of Life Sciences Hunan Normal University Changsha China

**Keywords:** Aβ42, Alzheimer's disease, *Drosophila*, intracellular amyloid, serine proteases, Yip7

## Abstract

Emerging evidence suggests that intraneuronal Aβ accumulation represents an early pathogenic event in Alzheimer's disease (AD), preceding extracellular plaque formation and neuroinflammatory responses. However, whether targeting intracellular Aβ can halt disease progression and how this can be achieved in vivo remain unknown. While investigating the brain transcriptional responses to Aβ pathology, we identify a neuroprotective role for the serine protease Yip7 in a *Drosophila* AD model. Neuronal overexpression of *yip7* alleviates multiple Aβ42‐induced deficits, including declines in locomotor activity, impaired proteostasis, increased brain aging and neuronal death, and shortened lifespan. Unlike canonical digestive proteases, Yip7 is not secreted but instead localized to the endosomal/lysosomal compartments via a putative transmembrane domain initially predicted as a signal peptide. Crucially, Yip7's neuroprotective function depends on its proper subcellular localization rather than the catalytic triad. Mechanistically, rather than eliminating Aβ, Yip7 binds intracellular Aβ42 to increase its neuronal retention, and this unexpectedly reduces Aβ42 toxicity to the organism. Finally, transcriptomics reveals that protection against Aβ42 toxicity by Yip7 is associated with selective suppression of Aβ‐upregulated genes including those codingribosomal proteins and molecular chaperones for protein folding. Together, these findings introduce a novel concept that intracellular sequestration of Aβ can be explored to mitigate its neurotoxicity.

## Graphical abstract

Emerging evidence suggests that intraneuronal Aβ accumulation represents an early pathogenic event in Alzheimer's disease (AD). Using *Drosophila* AD model, this study shows that a nonsecreted serine protease Yip7 physically interacts with Aβ. This causes intraneuronal Aβ accumulation but surprisingly reduces the associated neurotoxicity, arguing that sequestrating intracellular amyloids can be a possible therapeutic strategy for AD.

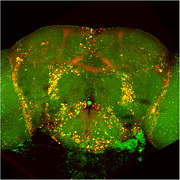

## 1 Introduction

Alzheimer's disease (AD), the predominant form of dementia, represents an urgent and escalating challenge worldwide. AD is a result of a complex interplay between genetic and environmental factors. Despite significant advances in identifying new biomarkers and understanding AD pathology and symptomatology, unfortunately, there is yet no cure for AD. Patients are frequently diagnosed at a late and irreversible stage, facing an average survival period of 4–8 years. Patients with AD exhibit a substantial accumulation of amyloid‐β (Aβ) plaques and neurofibrillary tangles in their brains, accompanied by a cascade of pathological processes including neuroinflammation, synaptic dysfunction, mitochondrial and bioenergetic disturbances, as well as vascular abnormalities [1].

Accumulation of Aβ has been widely accepted as a marker of AD. Since the amyloid cascade hypothesis, which posits that Aβ plaques cause AD, was introduced in 1992 [2], significant efforts and resources have been devoted to developing disease‐modifying drugs that target Aβ accumulation [1, 3]. Nevertheless, many investigational agents targeting Aβ, including earlier anti‐Aβ monoclonal antibodies, BACE inhibitors, and anti‐Aβ vaccines have demonstrated limited to no clinical benefits and variable side effect profiles [4]. Recently, lecanemab and donanemab, two monoclonal antibodies targeting cerebral Aβ plaques, led to slowing of cognitive and functional decline and positive alterations in disease‐specific biomarkers. Still, extensive debates exist centered on the efficacy, safety, and underlying evidence for the amyloid cascade hypothesis, exemplified by the recent rejection of lecanemab by the European Medicines Agency despite its approval in several other countries [5]. Moreover, AD pathological changes in the brain begin during the preclinical stage, decades before clinical symptoms appear [1]. Therefore, immediate early interventions to prevent pathogenic Aβ accumulation are still highly demanded, necessitating a re‐examination of Aβ etiology from a cell biology perspective.

Amyloid precursor protein (APP) is a single transmembrane protein. In the amyloidogenic pathway, APP is first cleaved by β‐secretase and the resulting C‐terminal fragment is subsequently cleaved by γ‐secretase to release Aβ [6]. APP processing and Aβ production do not occur simply on the cell surface, but rather involve complex intracellular protein sorting mechanisms via the endocytic and secretory pathways that remain not entirely understood [3, 7, 8]. Genetic studies have implicated endosome and lysosome trafficking in the pathogenesis of AD, along with other mutations altering APP processing, innate immunity particularly microglia activation, and lipid‐related processes [9, 10]. As endosomes are central players in membrane trafficking and abnormal enlargement of the early endosome has emerged as a cytopathological hallmark of AD, endosomal traffic jams and the associated intracellular accumulation of Aβ have been proposed as a pathogenic hub and a valid therapeutic target for AD drug discovery [11].

Indeed, Aβ was previously known to accumulate intraneuronally. In addition to intraneuronal Aβ production either in endosome compartments or along the secretory pathway, its reuptake from the extracellular space may also contribute to intracellular Aβ accumulation [7]. Although difficult to distinguish the in vivo source of intraneuronal Aβ, self‐produced and internalized Aβ peptides are both sorted via the endosomal/lysosomal system [12]. Notably, recent work revealed that intraneuronal Aβ accumulation can arise from impairments in the neuronal autophagy–lysosomal pathway [13, 14, 15, 16], and causally underlies selective neuronal vulnerability in AD [17, 18, 19]. Close dissection of mouse models and human AD pathology suggests that intracellular accumulation of Aβ in neurons precedes extracellular Aβ deposition and neuroinflammation elicited by microglial and astrocyte activation toward neuritic plaques [20, 21, 22, 23]. As chronic activation of these brain immune cells prunes neuronal synapses and compromises neuronal survival [12, 24, 25], intraneuronal Aβ appears as a significant immunological component in the initiation of AD pathogenesis. Therefore, targeted degradation or trapping of intracellular Aβ to block its toxicity would represent an immediate early strategy to reduce the subsequent neurotoxicity of Aβ.

Loss of proteostasis is causally involved in the pathogenesis of many neurodegenerative diseases [26]. Protein quality control by targeted degradation is usually carried out by the autophagy–lysosome and ubiquitin–proteasome systems [15, 27]. Nevertheless, these cellular sinks of unwanted proteins are often dysfunctional in AD. For example, Aβ42 can cause proteasome inhibition [28], and impair the endosomal–lysosomal pathway [29, 30], allowing Aβ to resist clearance by forming a self‐reinforcing detrimental cycle [6]. It is emerging that, proteases and peptidases, a large class of proteolytic enzymes with diverse substrate specificity and intracellular localization, can in principle act as an alternative route to degrade pathogenic proteins. Indeed, some proteases were reported to be induced as a protective response during proteasome dysfunction [31, 32], but the role of proteases in aging and neurodegeneration remains generally elusive [33]. Here, using *Drosophila* AD model [34, 35], we provide evidence that Yip7, a previously unstudied fly serine protease with some homology to human chymotrypsinogens (B1, B2, C, and L), can trap Aβ intracellularly in the late endosomes/lysosomes, but surprisingly reduces the neurotoxicity of Aβ peptides without directly eliminating them. This points to a novel avenue to target intracellular Aβ42 by sequestration and should inform alternative therapeutic strategies for AD.

## 2 Results

### 2.1 Transcriptomics of AD Flies Identified the Serine Protease Yip7

**FIGURE 1:**
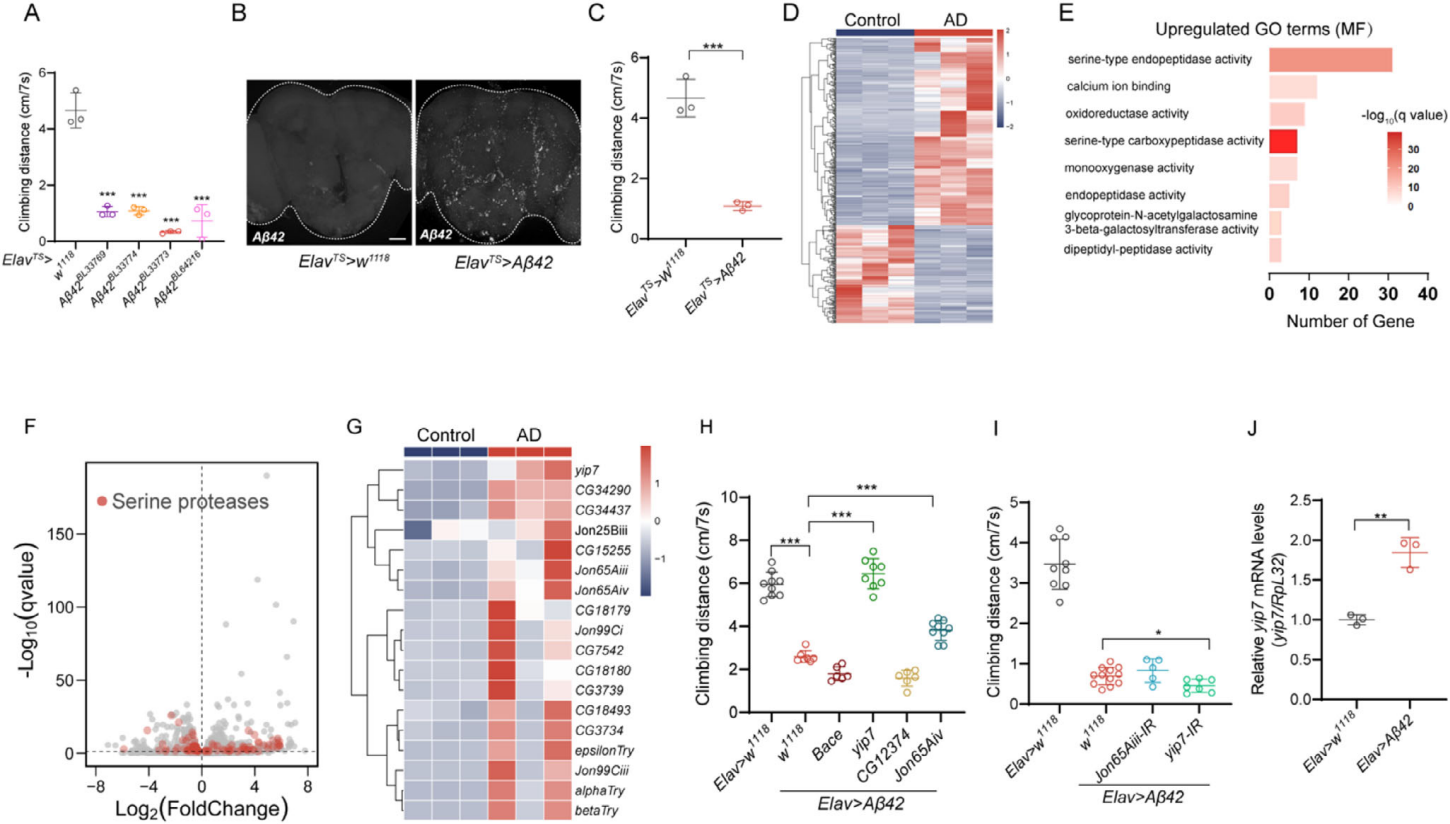
Transcriptomic analysis implicates the serine protease Yip7 in AD pathology. (A) Climbing ability of flies expressing the indicated *UAS‐Aβ42* transgenes pan‐neuronally with *Elav^TS^* (*Elav‐Gal4^c155^*; *tub‐Gal80^TS^*) for 14 days. (B,C) Aβ42 immunostaining (B) in the brains of flies with indicated genotypes reared at 29°C for 2 weeks, and their climbing ability (C). (D) A heatmap presentation of differentially expressed genes (*q* < 0.05 and Log_2_FC > 1 or < −1) from RNA‐seq comparing 14‐day‐old AD and control flies. Fly heads were used for RNA‐seq. (E,F) Gene ontology (GO) enrichment analysis (E) and volcano plot (F) of the differentially expressed genes (*q* < 0.05). Genes coding serine protease are highlighted in F. (G) A heatmap representing the expression profile of top upregulated genes involved in proteolysis (GO:0006508). (H,I) Climbing ability of flies overexpressing (H) or knocking down (I) the indicated protease/peptidase genes pan‐neuronally in AD flies (*Elav > Aβ42*) for 14 days. (J) qPCR measuring *yip7* mRNA levels in heads of control and AD flies. For climbing assays (A,C,H,I), the average distance climbed by 10 flies within 7 s was plotted as one dot. Each dot represents one independent experiment in A, C, and H–J. Statistical analyses were conducted using Student's *t*‐test to compare two groups, using one‐way ANOVA with Tukey's post hoc test for multiple comparisons. All data are presented as mean ± SD. ***p < 0.001; **p < 0.01; *p < 0.05; ns, not significant. Scale bar: 50 µm in B.

As there is no endogenous Aβ production in *Drosophila* [36], we adapted a widely used fly AD model by pan‐neuronally expressing the human Aβ42 peptides using *Elav‐Gal4^c155^* or *nSyb‐Gal4* as drivers [37]. After having tested several *UAS‐Aβ42* lines in locomotor assays (Figure 1A), we opted to further use a highly pathogenic form of human Aβ42 that carries the “Arctic” Swedish mutation (E22G in Aβ42, corresponding to E693G in APP) fused to the N‐terminal signal sequence of fly Argos for secretion [35, 38]. This Arctic Aβ42 was previously found to be more prone to form large deposits in the cell body and more pathogenic than wild type Aβ42 [38]. Flies expressing this line exhibited a rapid increase in Aβ42 accumulation in the brain and decline in locomotor activity (Figure 1B,C) and are referred to as AD flies. To investigate the mechanisms underlying AD pathogenesis, RNA sequencing (RNA‐seq) was performed to analyze the gene expression profile in the head of AD and control flies collected 14 days after Aβ42 transgene expression. This identified 395 significantly upregulated and 150 downregulated amyloid‐responsive genes with a cutoff at twofold (Figure 1D). Among these genes, *CG13397*, *ZnT86D*, and *LUBEL* were recently found in transcriptomic analyses of AD flies [39], confirming the efficacy of our dataset. Gene ontology (GO) analysis revealed a strong enrichment of serine proteases in upregulated genes (Figure 1E,F). These included many known fly digestive enzymes, the Jonah proteases and trypsins (Figure 1G). Given the potential role of proteases in regulating protein homeostasis, a process tightly relevant to AD pathogenesis [15], we focused on genes of this category.

Using climbing ability as a measurement of AD pathology, we performed a screen of proteases potentially involved in AD pathogenesis by overexpression or RNAi‐mediated depletion. This identified yippee interacting protein 7 (Yip7), a previously unstudied putative chymotrypsin‐like serine protease [40]. Strikingly, overexpressing *yip*7 completely restored the climbing ability of AD flies to the levels of wild type control flies, contrasted with other proteases that showed no (*Bace* (*beta‐site APP‐cleaving enzyme*) and *CG12374*, a putative metallocarboxypeptidase) or limited improvement (*Jon65Aiv*, a serine protease) (Figure 1H). On the other hand, Yip7 is also required in neurons for the climbing capacity of AD flies, as knocking down *yip7* but not *Jon65Aiii* led to further reduction in climbing (Figure 1I). Thus, the induction of *yip7* transcription by Aβ42, which we confirmed with qPCR (Figure 1J), appears as a protective physiological response of the organism to Aβ deposition, though not reaching a level sufficiently to block AD progression.

### 2.2 Overexpressing Yip7 Represses Aβ42‐Induced Deficits

Next, we systematically assessed the extent to which Yip7 expression can alleviate multiple AD‐associated deficits, including locomotor activity, proteostasis, neuronal death, and lifespan.

**FIGURE 2:**
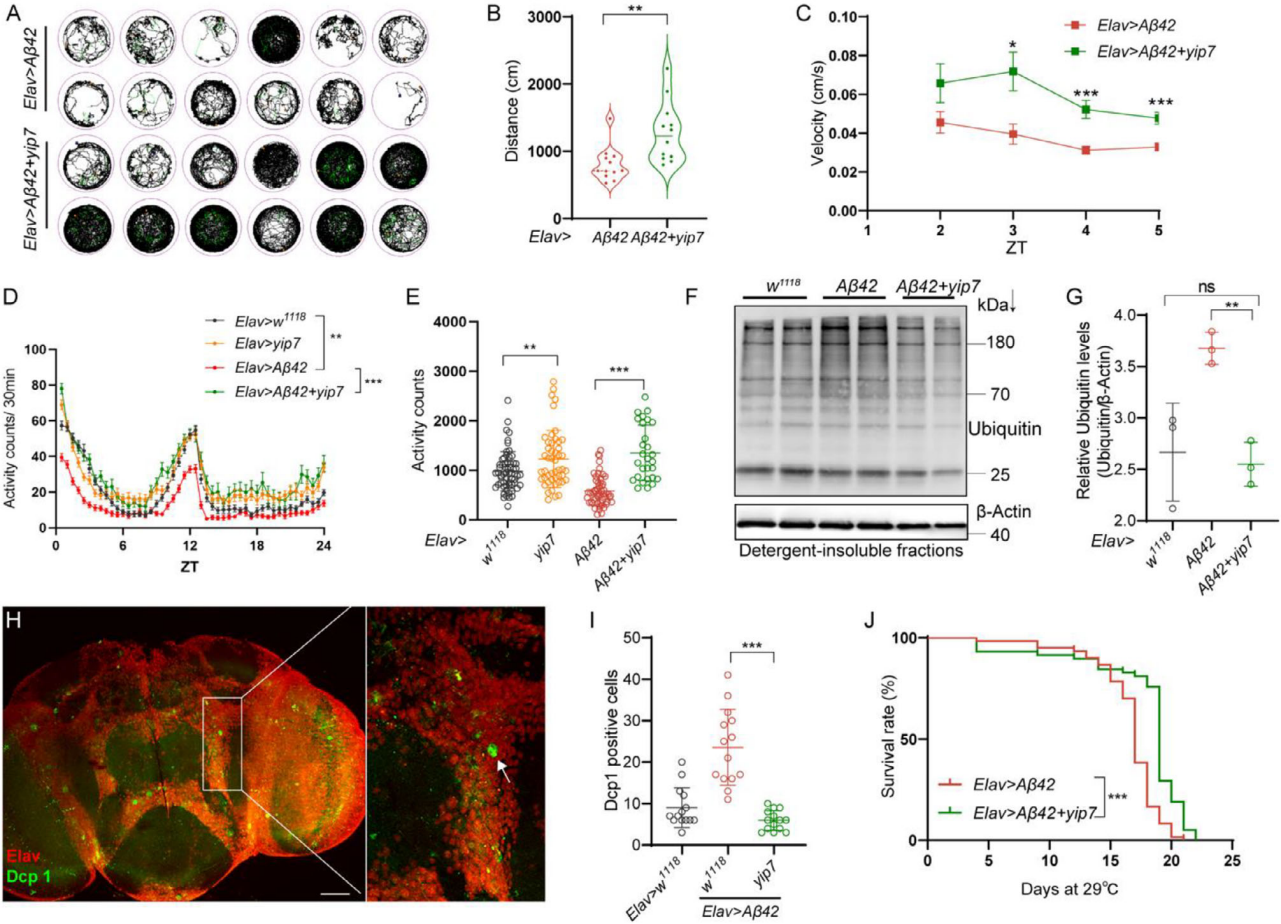
Yip7 suppresses Aβ neurotoxicity. (A–C) Trajectories (A) of spontaneous movements of *Elav* > *Aβ42* and *Elav* > *Aβ42* + *yip7* flies monitored with the ViewPoint Zebrabox tracking system for 5 h, and the quantification of the distance travelled (B) and the velocity (C). Each well contains a single fly in A; *n* = 12 flies for each genotype. (D,E) Activity plots over ZT times (D) and quantification of the total activity (E) of flies with the indicated genotypes analyzed with the *Drosophila* activity monitor (DAM) system. *n* = 32 flies for each genotype. (F,G) A representative blot (F) and gray scale quantification of the blot with Fiji (G) of poly‐ubiquitinated proteins in detergent‐insoluble protein fractions from heads of flies with the indicated genotypes reared at 29°C for 14 days. (H,I) A representative immunofluorescence image of Dcp1^+^ cells (H) and quantification of Dcp1^+^ cells in the central brains (I) of 14‐day‐old flies with the indicated genotypes reared at 29°C. Elav staining marks neurons; each dot represents one brain. (J) Lifespan analysis of the indicated flies at 29°C. *n* = 60 flies for each genotype. Statistical analyses were conducted using Student's *t*‐test to compare two groups, using one‐way ANOVA with Tukey's post hoc test for multiple comparisons, or using Log‐rank (Mantel–Cox) test for lifespan analysis. All data are presented as mean ± SD except lifespan. ***p < 0.001; **p < 0.01; *p<0.05; ns, not significant. Scale bar: 50 µm in H.

First, video tracking of spontaneous walking indicated that Yip7 expression significantly improved the locomotor performance of AD flies, with longer distance traveled at higher velocity (Figure 2A–C), whereas expressing *Jon65Aiv* or *Bace* did not (Figure S1A,B). *Drosophila* activity monitor (DAM) system was further used to track fly activity over a longer period of time. Expressing *Aβ42* or *yip7* in neurons did not change the overall daily activity pattern, but the reduction of activity counts in AD flies was completely reversed by coexpressing *yip7* (Figure 2D,E). These results show that neuronal Yip7 enhances spontaneous locomotor activity in AD flies.

Second, the dysfunction or failure of proteasomal degradation is associated with diverse human diseases including neurodegeneration [41]. We measured levels of detergent‐insoluble poly‐ubiquitinated protein aggregates and found that Yip7 expression effectively corrected Aβ‐induced impairment in protein quality control (Figure 2F,G). To explore whether Yip7 also mitigates the protein homeostasis disruption caused by other factors, we adapted a paraquat‐induced oxidative stress model [42, 43]. As previously reported [42], neuronal overexpression of the Hsp70 molecular chaperone increased fly survival to paraquat treatment. In contrast, pan‐neuronal *yip7* overexpression did not significantly change fly resistance to oxidative stress (Figure S1C). In addition, pan‐neuronal expression of *yip7* increased neither fly longevity (Figure S1D) nor protection against eye degeneration in disease models of amyotrophic lateral sclerosis (ALS) [44] (Figure S1E). Thus, Yip7 appears to more specifically protect against pathogenic Aβ42‐induced neurotoxicity, without conferring broader protection against stresses.

Furthermore, Aβ deposition triggers neuronal death and mortality [36]. Strikingly, the increased neuronal cell death in the brains of AD flies marked by the activation of Dcp1, a caspase‐3‐like effector caspase, was strongly reduced by expressing *yip7* (Figure 2H,I). Finally, *yip7* overexpression extended the lifespan of AD flies (Figure 2J). Collectively, these findings demonstrate a prominent role of the Yip7 protease in protecting against Aβ neurotoxicity.

### 2.3 Yip7 Is Not Secreted but Localized to Endosomal/Lysosomal Compartments in Neurons

**FIGURE 3:**
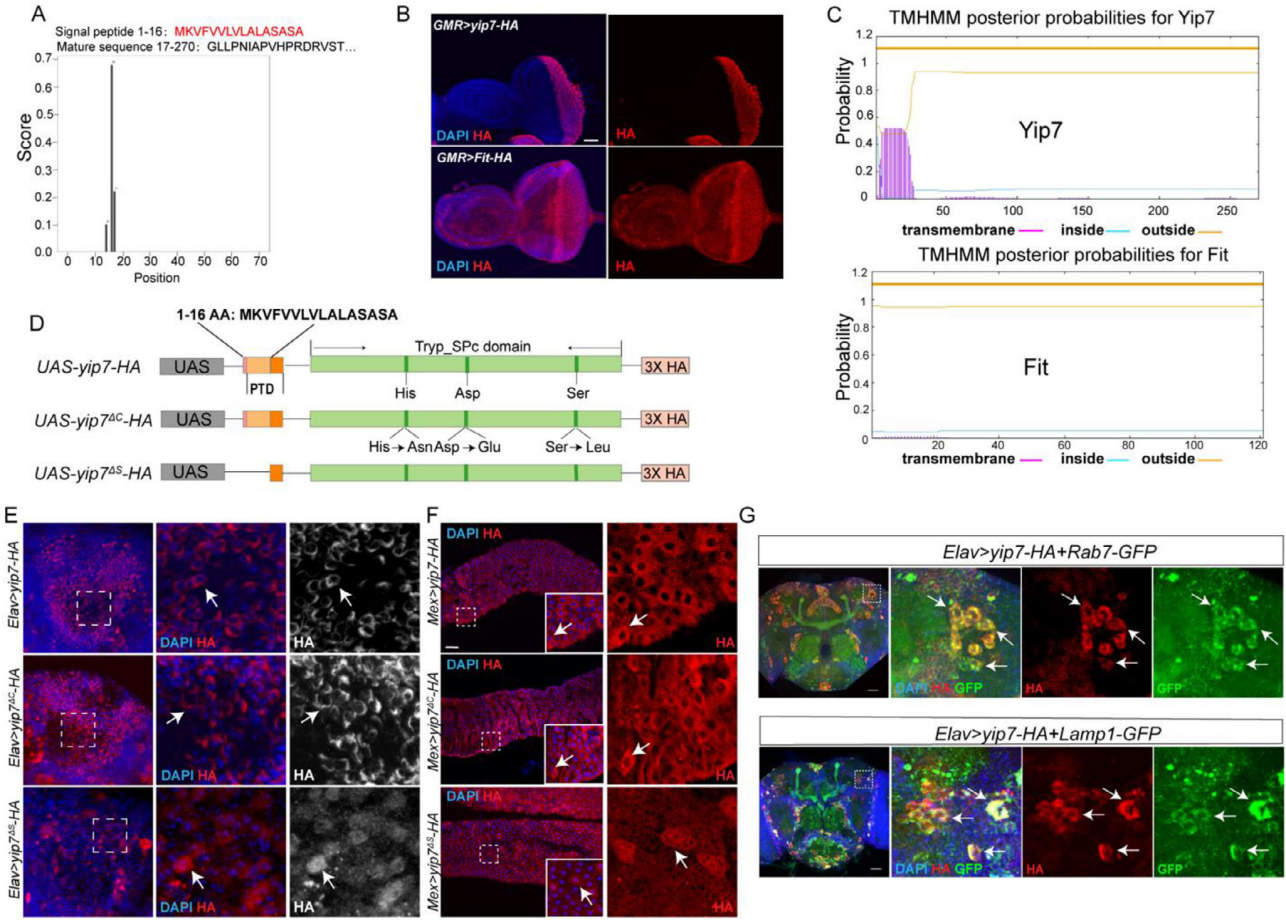
Subcellular localization of Yip7. (A) Signal peptide prediction for Yip7 protein using Signal‐3L 3.0. (B) Immunostaining detecting the localization of HA in the eye‐antenna discs of third‐instar larvae expressing either *UAS*‐*Yip7*‐*HA* or *UAS*‐*Fit*‐*HA* in the eye proportion driven by *GMR‐Gal4*. Note that Yip7‐HA was found only in the differentiating photoreceptors while Fit‐HA was distributed throughout the eye‐antenna discs. (C) Transmembrane domain prediction for Yip7 and Fit using TMHMM 2.0. (D) Illustration of protein features of Yip7 and Yip7 variants used to generate transgenic lines. Amino acids 1–16 were predicted to be a signal peptide, but residues 3–25 also exhibit features of a potential transmembrane domain (PTD). Trypsin‐like serine protease (Tryp_SPc) domain is depicted in green with the catalytic triad residues (His–Asp–Ser) indicated. (E,F) Expression of the different Yip7‐HA variants in the mushroom body (E) and the midgut epithelium (F). HA (red) and DAPI (blue) are shown. (G) Yip7‐HA colocalization with organelle markers. Rab7‐GFP for late endosomes and Lamp1‐GFP for lysosomes. Scale bars: 50 µm for all the images.

We further sought to reveal protein features of Yip7 underlying its neuroprotective role. Yip7 appears a regular serine protease from prediction based on its protein sequence, with the first 16 residues at the N‐terminus serving as a signal peptide for secretion (see also prediction by Signal‐3L 3.0 [45], Figure 3A) and carrying the typical chymotrypsin‐like serine protease domain with the classic His–Asp–Ser catalytic triad [40, 46]. However, immunostaining showed that Yip7‐3xHA protein expressed in neurons was found in the cytoplasm, possibly within certain intracellular membranous organelles as suggested by the shape of HA staining. Yip7‐3xHA protein expressed in enterocytes was also found in the cytoplasm. To verify whether Yip7 is indeed not secreted, we used *GMR‐Gal4* to express transgenes in the eye imaginal discs and checked the localization of the expressed protein (Figure 3B). While Fit (*UAS*‐*Fit*‐*HA*), previously known to be secreted [47], diffused anteriorly and accumulated throughout the whole eye‐antenna imaginal disc, Yip7‐3xHA was restricted to the posterior part of the eye disc, in cells undergoing differentiation into the mature retina (i.e., regions with *GMR‐Gal4* expression). We thus conclude that Yip7 protein is, in fact, not secreted.

Interestingly, the region from the 3rd to the 25th residues partially overlapping with the predicted signal peptide shows features of a transmembrane domain as predicted by TMHMM 2.0 [48] (Figure 3C). To check if the localization of Yip7 depends on this putative transmembrane domain (PTD), we generated UAS transgenes expressing truncated Yip7 lacking this PTD (*UAS‐yip7^∆S^‐3xHA*). Two additional transgenes of Yip7 expressing either wild type Yip7 (*UAS‐yip7‐3xHA*) or Yip7 with a mutated catalytic triad (*UAS‐yip7^∆C^‐3xHA*) were also created (Figure 3D). As the three transgenes were constructed in the same way and integrated into the same genomic locus, their localization and functions can be directly compared. By crossing these transgenes to Gal4 drivers, we found that the proper cytoplasmic localization of Yip7 requires the PTD but not the catalytic triad, in both neurons and gut epithelial cells (Figure 3E,F). To determine the subcellular location of Yip7, we applied a panel of markers for organelles including the ER, Golgi, mitochondria, nuclear lamina, and different endosomal/lysosomal compartments (early endosomes, late endosomes, and the lysosomes), as well as markers for neuronal secretion, including syt‐GFP indicative of synaptic versicles and ANF‐GFP that marks dense‐core vesicles for neuropeptide release (Figure 3G; Figure S2). Close examinations identified an extensive colocalization of Yip7 with Rab7 (marking late endosome) and Lamp1 (marking the lysosome) in neurons (Figure 3G). Interestingly, the F–V–V–L motif present at the N‐terminus of Yip7 is reminiscent of the LC3‐interacting motif that ensures protein targeting to LC3 anchored in the phagophore membrane, a structure that subsequently matures by recruiting lysosomes [49]. Together, these results show that a cryptic transmembrane domain is required to direct Yip7 to the endosomal/lysosomal compartments.

### 2.4 Yip7 Physically Interacts With Aβ42 and Promotes Its Intracellular Accumulation

**FIGURE 4:**
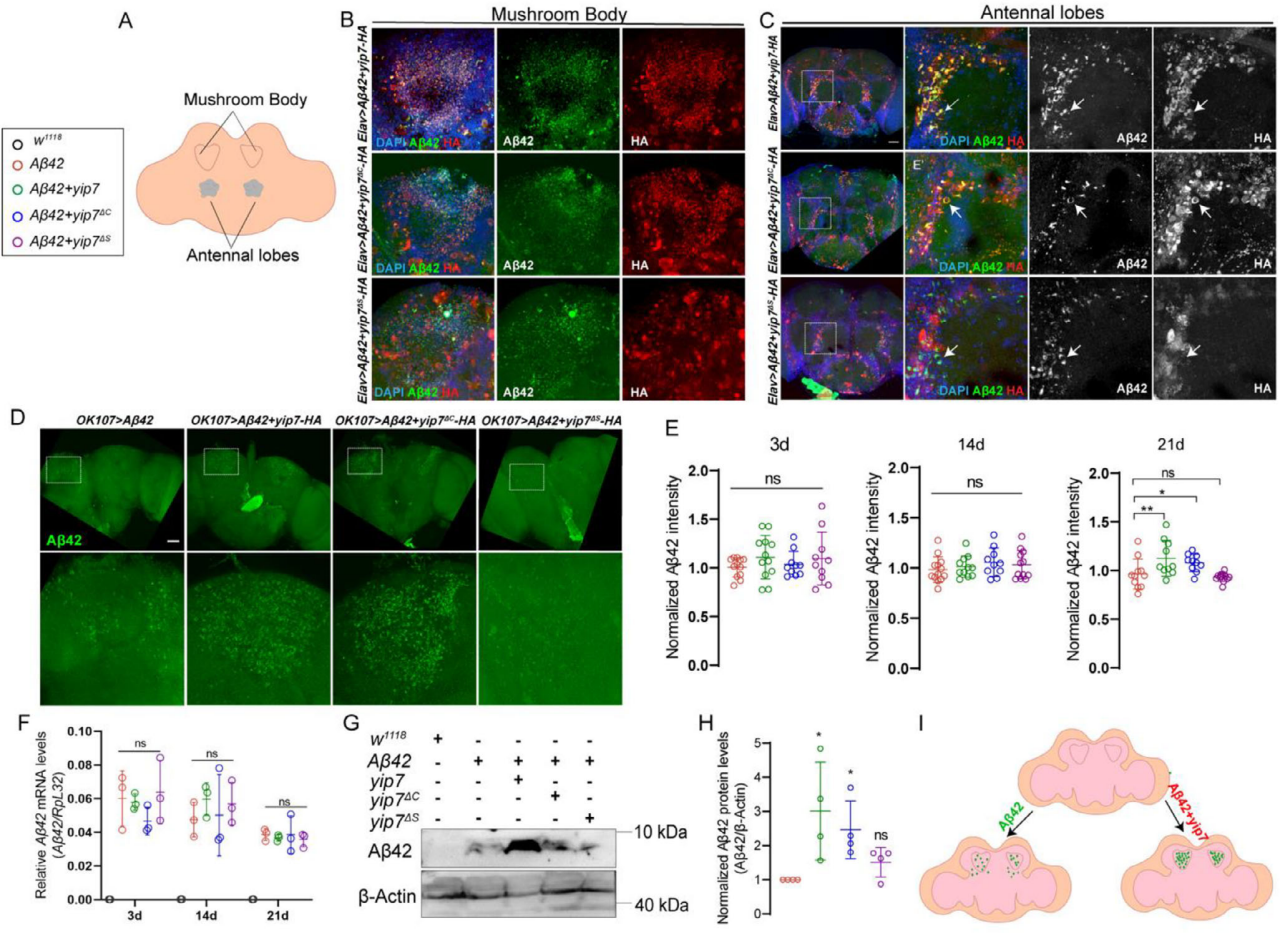
Yip7 promotes intracellular Aβ accumulation. (A) Illustration of the two brain regions that this study has focused on to reveal Aβ42 localization and accumulation: mushroom bodies and antennal lobes. (B,C) Representative images showing colocalization of Yip7‐HA variants with Aβ42 in mushroom bodies (B) and in antennal lobes (C). (D) Representative images of Aβ42 staining in flies expressing *Aβ42* alone or simultaneously coexpressing *yip7*, *yip7* ^Δ^ *^C^*, or *yip7* ^Δ^ *^S^* in mushroom bodies using *OK107‐Gal4* for 21 days at 29°C. (E) Quantification of relative Aβ42 intensity in mushroom bodies over time. Note that Aβ42 intensity was normalized to 1 for each time points. Each dot indicates one brain. (F) qPCR analysis of *Aβ42* mRNA levels in heads of flies with the indicated genotypes raised at 29°C for 3, 14, and 21 days. Each dot indicates one biological replicate. (G,H) A representative blot and quantification of Aβ42 protein levels in heads of flies coexpressing Aβ42 with one of the three Yip7 variants pan‐neuronally using *nSyb‐Gal4*. Each dot indicates one biological replicate in H. (I) Schematic diagram illustrating that Yip7 inhibits neurotoxicity by limiting Aβ42 secretion. Color‐coded genotypes as indicated in the text box apply to all quantifications in E, F, and H. Statistical analyses were conducted using one‐way ANOVA with Tukey's post hoc test. All data are presented as mean ± SD. **p < 0.01; *p < 0.05; ns, not significant. Scale bars: 25 µm in B and 50 µm in C and D.

The endosomal–lysosomal system is a key route for vesicle trafficking, protein sorting, and targeted degradation of unwanted proteins, and is emerging as a central player in neurodegenerative diseases [50]. Initially, we hypothesized that, as an endosome/lysosome‐localized serine protease, Yip7 is ideally positioned for Aβ degradation through proteolysis. Intriguingly, Yip7 was found to be perfectly colocalized with Aβ42 in neurons (Figure 4A–C). By contrast, another serine protease Jon65Aiv that lacks this PTD, did not colocalize with Aβ42 (Figure S3A). As expected, the ability of Yip7 to colocalize with intracellular Aβ requires the PTD for proper protein targeting, but not the catalytic triad (Figure 4A–C).

To probe how Yip7 impacts Aβ, we turned to the *OK107‐Gal4* driver that is expressed in a more restricted neuronal population, the Kenyon cells of the mushroom body (MB) [51]. By driving Aβ42 expression with this driver, we could readily quantify Aβ protein levels by imaging the MB calyx [38]. Strikingly, rather than reducing Aβ levels, expressing *yip7* in AD flies led to a significant neuronal retention of Aβ (Figure S3B,C). While *OK107* > *Aβ42* + *Yip7* flies did not show a significant increase in Aβ levels at 3 and 14 days, 21‐day‐old flies exhibited a significantly higher level of Aβ accumulation than *OK107* > *Aβ42* flies (Figure 4D). Again, retention of Aβ in the MB calyx required the PTD that is required for Yip7 subcellular localization (Figure 4E). Furthermore, Aβ accumulation was still maintained at a high level upon expressing the catalytic dead mutant of the Yip7 protease, indicating that the proteolytic activity of Yip7 is not involved in Aβ sequestration (Figure 4D,E). Thus, Yip7 likely acts as a scaffold protein rather than a protease. Further supporting a role of Yip7 in trapping intracellular Aβ, the transcript levels of *Aβ* did not differ between flies expressing *Aβ* alone or in combination with one of the three *yip7* transgenes (Figure 4F), but Western blots showed that the total Aβ protein levels in the heads were increased in flies coexpressing either wild type Yip7 or the catalytically dead mutant Yip7^∆C^, but not in flies coexpressing the protein‐targeting mutant Yip7^∆S^ (Figure 4G,H). This implies that Aβ trapped intracellularly by Yip7 has avoided degradation either by the protein quality control machinery or due to reduced release into the extracellular space (Figure 4I). Consistent with this notion, it has been shown that extracellular Aβ peptides are removed by glial engulfment [35]. In addition, Aβ retention by Yip7 was also seen in the insulin‐producing cells (Figure S3D), neurons located in the pars intercerebralis region [52]. We note that pan‐neuronally expressed Yip7^∆S^‐3xHA protein was detected at lower levels than Yip7‐3xHA and Yip7^∆C^‐3xHA in Western blots, suggesting reduced protein stability due to a failure of proper protein targeting (Figure S3E).

**FIGURE 5:**
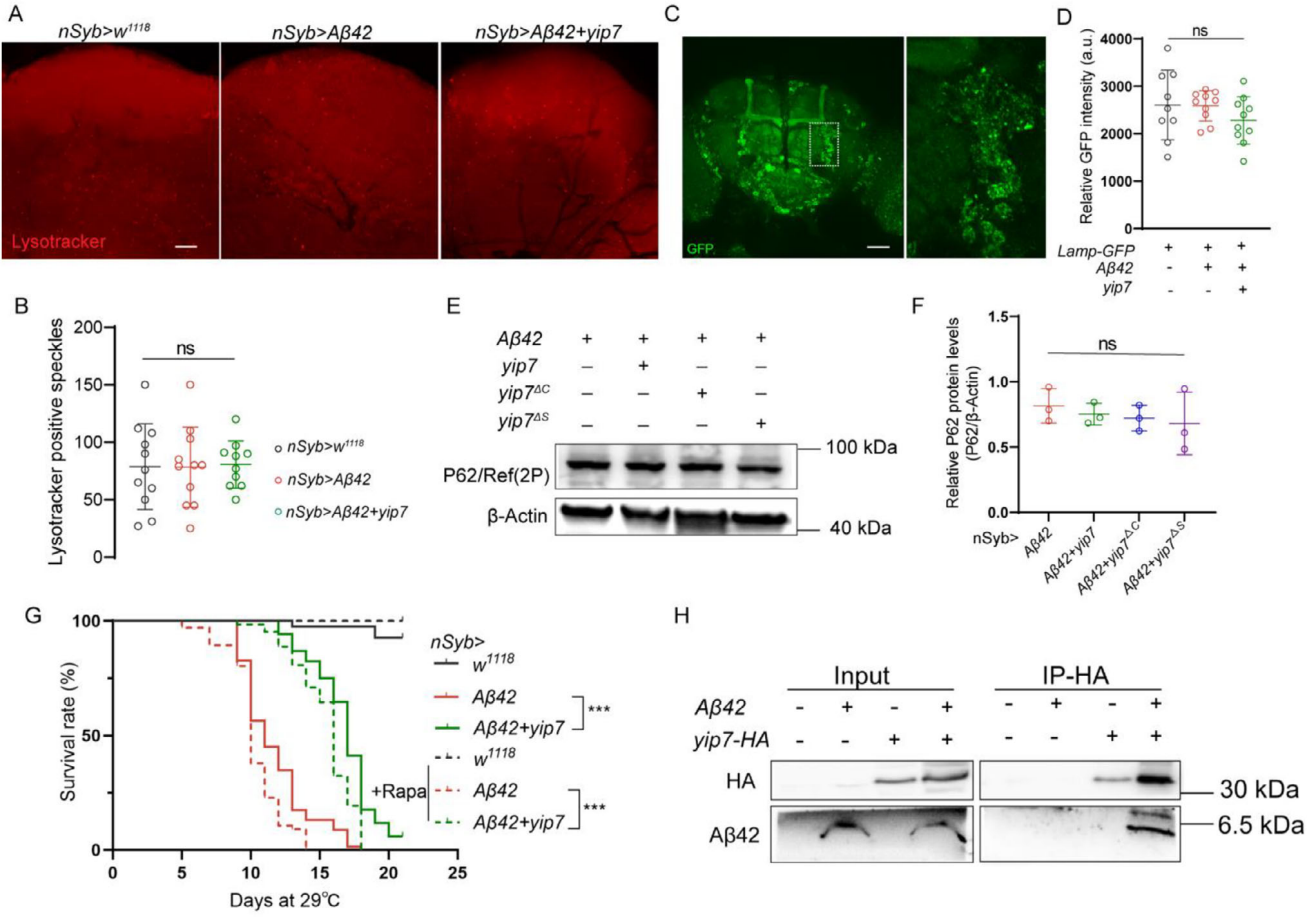
Yip7 physically interacts with Aβ42. (A,B) Representative images of LysoTracker staining in the brain region of interest (the mushroom body) (A) and quantification of the LysoTracker‐positive puncta (B) in control flies, flies expressing *Aβ42* alone or simultaneously coexpressing *yip7* pan‐neuronally using *nSyb‐Gal4* for 10 days at 29°C. Each dot indicates one brain in B. (C,D) The region of interest (brain regions flanking the antenna lobes) where staining of Lamp‐GFP driven by *nSyb‐Gal4* was imaged (C) and quantification of Lamp‐GFP staining intensity (D) in flies with the indicated genotypes raised at 29°C for 10 days. Each dot indicates one brain in D. (E,F) A representative Western blot (E) and Fiji quantification (F) of P62/Ref(2P) levels in the heads of flies with the indicated genotypes raised at 29°C for 10 days. Each dot indicates one biological replicate in F. (G) Lifespan of *nSyb* > *w^1118^*, *nSyb* > *Aβ42*, and *nSyb* > *Aβ42* + *yip7* flies raised at 29°C with or without rapamycin (1 µm) supplementation. *n* > 60 flies for each sample. (H) in vivo co‐immunoprecipitation (co‐IP) using heads of flies with the indicated genotypes (driven by *nSyb‐Gal4*) raised at 29°C for 10 days. Note that Yip7‐HA and Aβ42 physically interact. Representative blots of three independent replicates are shown. Statistical analyses were conducted using one‐way ANOVA with Tukey's post hoc test in B, D, and F, or using Log‐rank (Mantel–Cox) test for lifespan analysis in G. All data are presented as mean ± SD except lifespan. ***p < 0.001; ns, not significant. Scale bars: 24 µm in A and 50 µm in C.

We then tested the possibility that Yip7 impairs the lysosomal function or autophagy and in turn causes intracellular Aβ accumulation. Several lines of evidence argued against this notion. First, LysoTracker staining and the expression of a Lamp1‐GFP reporter were not changed by Yip7 (Figure 5A–D). Second, p62 (Ref(2)P in *Drosophila*) marks ubiquitinated protein bodies for autophagic degradation, and accumulates in cells during inhibition of autophagy [53]. Nevertheless, we failed to detect any difference in p62 levels between flies expressing Aβ alone and those simultaneously expressing Yip7, Yip7^∆C^ or Yip7^∆S^ (Figure 5E,F). Third, rapamycin treatment that inhibits mTORC1 activity and in turn activates autophagy and lysosomal function in both flies and mammals [54] similarly shortened the lifespan of flies pan‐neuronally expressing Aβ and flies simultaneously coexpressing Yip7 (Figure 5G), even though rapamycin supplementation is known to provide health benefits to a range of wild type animals and disease models [55, 56, 57]. These datasuggest that Yip7 promotes neuronal retention of Aβ42, but unlikely through impairing lysosomal function or autophagy.

Finally, we determined if Yip7 physically associates with Aβ42 using an in vivo immunoprecipitation assay. Intriguingly, we consistently detected a direct physical interaction between Yip7 and Aβ42 (Figure 5H). Moreover, Yip7 seems to increase Aβ42 oligomerization, as an 8.5 kDa band indicative of Aβ42 dimers was detected in the Yip7‐bound Aβ42 fraction. Thus, our data are consistent with a model in which the endosome/lysosome‐targeted Yip7 protein encounters Aβ along its trafficking route, binds it and traps it inside the neurons (Figure 4I). Consistent with this notion, the presence of Yip7 did not change the overall staining pattern of intracellular Aβ (Figure 4D; Figure S3B), indicating that Yip7 does not alter the Aβ trafficking route but rather plays a sequestrating role, likely by suppressing Aβ intracellular degradation and/or Aβ secretion into the extracellular space. Somewhat unexpectedly, such intraneuronal Aβ sequestration limits its neurotoxicity.

### 2.5 Intraneuronal Retention of Aβ by Yip7 Blocks Aβ Neurotoxicity

**FIGURE 6:**
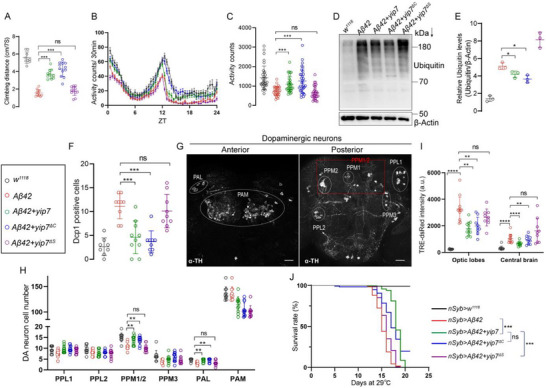
Promoting intracellular Aβ accumulation can counteract AD. (A) Climbing ability of flies expressing the indicated transgenes pan‐neuronally with *Elav‐Gal4^c155^* for 14 days. The average distance climbed by 10 flies within 7 s was plotted as one dot. Each dot indicates an independent test. (B,C) Activity plots over ZT times (B) and quantification of total activity (C) of flies with the indicated genotypes analyzed with the *Drosophila* Activity Monitorsystem. *n* > 30 flies for each genotype. (D,E) A representative blot (D) and gray scale quantification of the blots with Fiji (E) of poly‐ubiquitinated proteins in detergent‐insoluble protein fractions from heads of flies with the indicated genotypes reared at 29°C for 10 days. Each dot indicates one biological replicate in E. (F) Quantification of Dcp1^+^ cells in the central brain of 10‐day‐old flies with the indicated genotypes reared at 29°C. (G) Overview of the anterior (PAL and PAM) and posterior (PPM1/2, PPM3, PPL1, and PPL2) protocerebral dopaminergic (DA) neuron clusters in wild type flies by anti‐TH immunostaining. (H) Quantification of DA neuron numbers in six distinct clusters in flies with the indicated genotypes raised at 29°C for 10 days. (I) Quantification of TRE‐dsRed fluorescence intensity in the optic lobes (*left*) and central brain (*right*) of flies with the indicated genotypes raised at 29°C for 10 days. (J) Lifespan analysis of the indicated flies at 29°C. *n* > 100 flies for each genotype. Each dot represents one brain in F, H, and I. Color‐coded genotypes as indicated in the text box apply to all quantifications except J. *Elav‐Gal4^c155^* was used in A–C, and *nSyb‐Gal4* was used in D–J. Statistical analyses were conducted using one‐way ANOVA with Tukey's post hoc test for multiple comparisons and Log‐rank (Mantel–Cox) test for lifespan analysis. All data are presented as mean ± SD except lifespan. ****p < 0.0001; ***p < 0.001; **p < 0.01; *p < 0.05; ns, not significant. Scale bars: 50 µm in G.

We further extended our analyses of Yip7 variants in their ability to counteract Aβ neurotoxicity. Expressing wild type Yip7 and the catalytically dead Yip7^∆C^, but not the protein‐targeting mutant Yip7^∆S^ significantly suppressed the Aβ‐induced deficits in negative geotaxis as measured by a climbing assay (Figure 6A), in locomotor activity during spontaneous walking as measured with the DAM system (Figure 6B,C), in the accumulation of detergent‐insoluble poly‐ubiquitinated proteins (Figure 6D,E), and in the number of Dcp1^+^ cells (Figure 6F). Dopaminergic neurons (DANs) are associated with locomotion and other movements, and are frequently lost in animal models of neurodegenerative diseases [58, 59, 60, 61, 62]. Several DAN clusters can be visualized using an anti‐TH antibody in both the posterior protocerebrum (PPM1/2, PPM3, PPL1, and PPL2) and the anterior protocerebrum (PAL and PAM) [63]. Notably, DANs in the PPM1/2 cluster are tightly associated with locomotor functions. We found that expressing Aβ42 pan‐neuronally with *nSyb‐Gal4* significantly reduced the number of DANs in the PPM1/2 and PAL clusters (Figure 6G,H). Consistent with the other parameters measured above, expressing Yip7 and Yip7^∆C^, but not Yip7^∆S^, suppressed this reduction in DAN number (Figure 6G,H). Moreover, Aβ can accelerate senescence of the adult brain [64, 65]. It was recently shown that the senescence‐associated transcription factor AP1 is activated during aging and brain injury in the fly brain, including in glial cells [66]. Using the same *TRE‐dsRed* reporter that indicates AP1 activity as a measure of brain senescence, we found that expressing Aβ42 in neurons significantly induced AP1 activity in both the optic lobes and the central brain. As expected, expressing Yip7 and Yip7^∆C^, but not Yip7^∆S^ alleviated Aβ42‐induced AP1 activity, indicative of reduced brain senescence (Figure 6I; Figure S4A–E). Consistently, both Yip7 and Yip7^∆C^ extended the lifespan of AD flies, while Yip7^∆S^ had no effect (Figure 6J). These data support that intraneuronal retention of Aβ by Yip7 effectively blunts various Aβ‐induced pathologies.

### 2.6 Molecular Mechanisms Underlying Yip7's Protection Against Aβ

**FIGURE 7:**
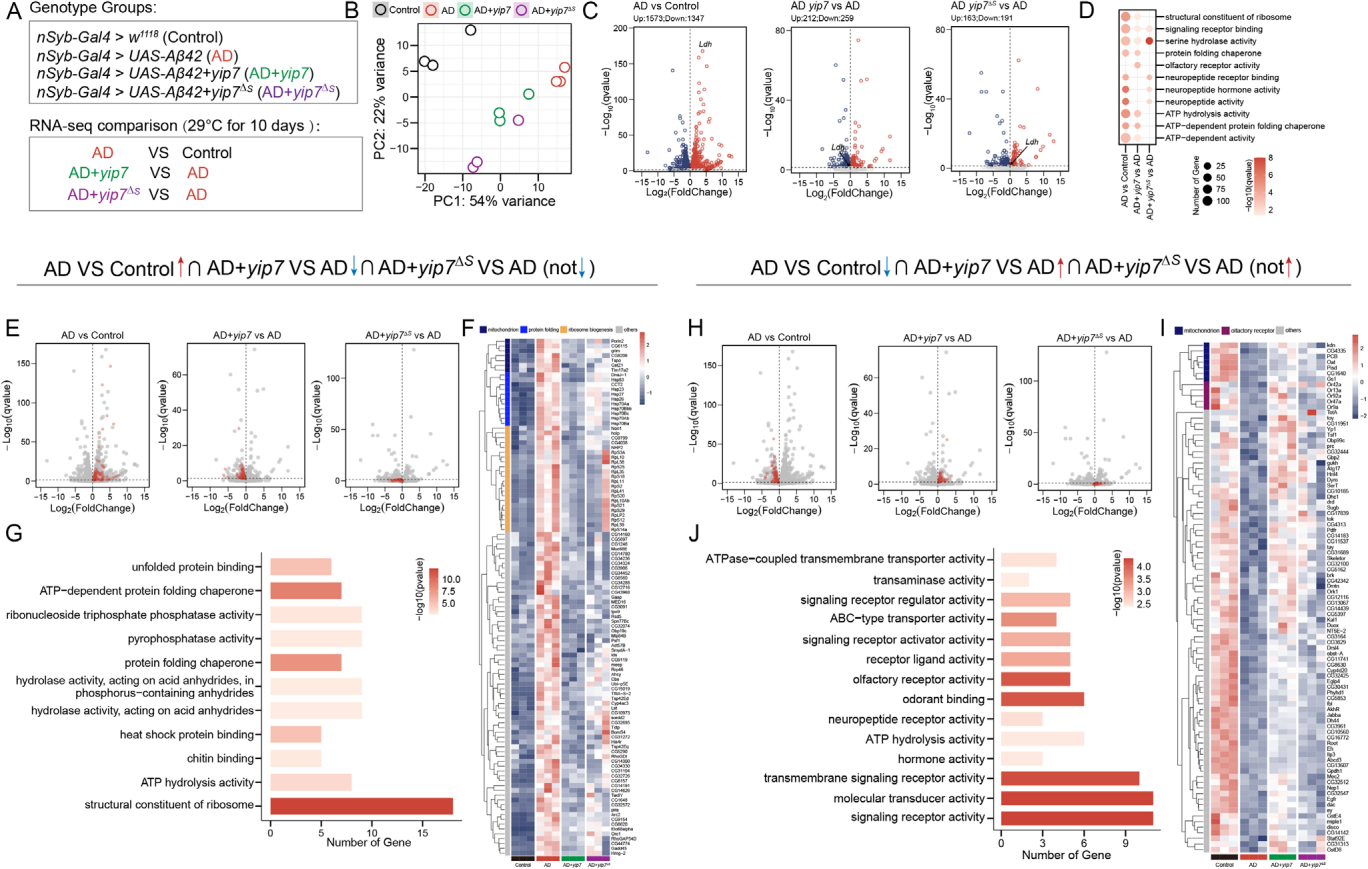
Transcriptomics to identify molecular mechanisms by which Yip7 protects against Aβ. (A) Experimental design of the transcriptomic study. Twenty fly heads per replicate were used for gene expression profiling in biological triplicates. (B) PCA analysis of RNA‐seq data. (C) Volcano plots showing differentially expressed genes (*q* < 0.05) in the three comparisons: AD vs. Control, AD + Yip7 vs. AD, and AD + Yip7^∆S^ vs. AD. The numbers of significantly upregulated and downregulated genes are indicated. *Ldh* is also marked. (D) GO enrichment in the three comparisons mentioned above. Selected GO terms (molecular function (MF); *q* < 0.05) significantly enriched between different comparisons are shown. (E–G) Volcano plots (E), heatmap of expression profiles (F) and GO terms enriched (G) for genes whose expression is significantly upregulated in AD and suppressed by coexpressing Yip7 but not by Yip7^∆S^. (H–J) Volcano plots (H), heatmap of expression profiles (I) and GO terms enriched (J) for genes whose expression is significantly downregulated in AD and induced by coexpressing Yip7 but not by Yip7^∆S^. The full lists of gene expression value and enriched GO terms are included in Table S1.

Transcriptomic analyses were again performed to uncover mechanisms by which Yip7 suppresses Aβ pathology (Figure 7A,B; Table S1). Although expressing Aβ42 pan‐neuronally for 10 days significantly changed the expression of 2920 genes (*q* < 0.05), coexpressing Yip7 or Yip7^∆S^ each altered only around 400 genes in AD flies (Figure 7C), suggesting that Yip7 did not globally reshape Aβ42‐related transcriptome. GO enrichment shows that among the four top processes (molecular function, MF) dysregulated in AD flies compared to wild type control (namely “structural constituent of ribosome”, “signaling receptor binding”, “serine hydrolase activity”, and “protein folding chaperone”), only those genes annotated as “structural constituent of ribosome” and “protein folding chaperone” were selectively altered by coexpressing Yip7 but not by Yip7^∆S^ (Figure 7D; Table S1). In contrast, the majority of Aβ42 changed genes (2601 out of 2920 genes) were not significantly altered by coexpressing Yip7, a condition that led to increased intracellular Aβ retention yet without causing greater neurotoxicity. A closer examination of genes in this category identified that the expression of many activity indicators of tissue inflammation and stress response was indeed induced by Aβ but not further increased by coexpressing Yip7 (Figure S5A,B). These included two fly NFkB transcription factors Relish (*Rel*) and Dorsal (*dl*), the AP1 transcription factor Fos (*kay*) and a widely used JNK stress pathway activity readout (the JNK phosphatase *puc*), *Socs36E* that reports cytokine‐induced JAK/Stat signaling activity, a stress‐associated MAPKKK (*Mekk1*) and a stress‐inducible transcription factor *Ets21C*, and several genes indicating ER stress (*Hsc70‐3*, *CaBP1*, *PHGPx*, *ERp60*, and *Xbp1*). Furthermore, our bulk RNA‐seq also identified a strong upregulation of *Lactate dehydrogenase* (*Ldh*) in AD flies, consistent with the recent AD Fly Cell Atlas data created with single‐nucleus transcriptomes [67]. Nevertheless, the high *Ldh* levels in AD flies were not suppressed by coexpressing Yip7, adding to the specificity of Yip7 action. In addition, the expression of cytokine‐like secreted factors (*egr*, *spz*, and *upd1‐3*) and many Toll effectors in host defense (e.g., *Bomanin* and *Drs*) did not follow a clear pattern in our dataset (Figure S5C). Collectively, these gene expression changes are consistent with the notion that the intracellular Aβ retention by Yip7 was not associated with increased brain inflammation.

We further focused on these Aβ‐regulated genes whose expression is reversed by Yip7 but not by Yip7^∆S^. First, a closer look at the Aβ‐induced genes that were significantly downregulated only by Yip7 but not by Yip7^∆S^ (107 genes in total) revealed a strong enrichment in genes coding ribosomal proteins (22 genes) and molecular chaperones for protein folding (11 genes) (Figure 7E–G). Of note, an aberrant upregulation of ribosome complexes was recently observed in AD patients [68]. In addition, the upregulation of molecular chaperones, as also reported by the AD Fly Cell Atlas project [67], likely occurred following proteostatic stress under conditions where protein folding and degradation pathways are overwhelmed or dysfunctional, since heat shock proteins have demonstrated roles against intracellular amyloids [69]. The selective suppression of these two groups of genes amongst the 1573 Aβ‐induced genes (Figure 7C) is striking, and further suggests that Yip7 masks Aβ toxicity mainly by maintaining proteostasis in the brain (see Figure 6D). Supporting this notion, Yip7 also suppressed the concerted upregulation of *Ahcy* (coding the S‐adenosylhomocysteine hydrolase) and *Cbs* (coding the cystathionine β‐synthase) in AD flies that promotes the transsulfuration branch of the methionine cycle and has been implicated in ER stress [70, 71]. The small set of genes selectively repressed by Yip7 (Figure 7F) thus offers feasible targets to be tested for their involvement in AD pathogenesis.

Second, the Aβ‐downregulated genes that were significantly induced only by Yip7 but not by Yip7^∆S^ (91 genes in total) included several genes encoding olfactory receptors (Figure 7H–J). This finding is in line with the observation that the sensory system is particularly vulnerable to Aβ42 toxicity [67], and in turn validates the neuroprotective effects of Yip7. A similar restoration of Aβ‐repressed genes implicated in neuronal functions (*AkhR*, *CG32547*, *Dh44*, *Eh*, *Ilp3*, *Pdfr*, and *SerT*) was also observed (Figure 7I). Intriguingly, Yip7 but not Yip7^∆S^ restored the expression of many genes coding mitochondrial metabolic enzymes, including *CG1640* (*Alanine aminotransferase*), *CG4335* (*Trimethyllysine hydroxylase*), *Gs1* (*Glutamine synthetase*), *Oat* (*Ornithine aminotransferase*), *Pisd* (*Phosphatidylserine decarboxylase*), and notably *kdn* (*Citrate synthase*) and *PCB* (*Pyruvate carboxylase*), two crucial enzymes involved in the tricarboxylic acid (TCA) cycle (Figure 7I). This implies an interesting possibility that Yip7 may prevent Aβ‐induced mitochondrial dysfunction. Among other genes in this group, *Dyro* has been identified as a suppressor in Aβ toxicity [72], and the increase in *Atg17* [73] and *Hnf4* [74] hints at changes in autophagy and lipid metabolism, respectively, which are highly relevant to AD (Figure 7I).

Collectively, our transcriptomic analyses not only consolidate the neuroprotective role of Yip7 against Aβ but alsounlock fundamental molecular processes that could serve as valid targets for developing anti‐AD therapy.

## 3 Discussion

*Drosophila* has been widely used as an in vivo model to dissect the mechanisms underlying Aβ toxicity, and has significantly advanced our understanding of the molecular features leading to Aβ toxicity [38, 75, 76, 77], Aβ production and trafficking [78, 79, 80, 81], the implication of protein quality control mechanisms [30, 35, 69, 82] and the microbiota [83] in AD progression, and how Aβ disrupts sleep and memory [84, 85]. In this study, we identified the serine protease Yip7 that effectively alleviates Aβ neurotoxicity using a fly AD model, and show that the targeting of Yip7 to the endosomal/lysosomal compartments is essential for its neuroprotective effects. Our findings that Yip7 selectively altered only a small subset of Aβ‐responsive genes highlights specific molecular processes that are essential for AD pathogenesis.

Since the introduction of the amyloid cascade hypothesis that posits that Aβ plaques cause AD [2], research has been focused on reducing the extracellular Aβ, but these efforts have appeared largely unsuccessful in drug development [1]. Recent studies support that Aβ plaques may originate inside the neuron, rather than in extracellular space as had been believed for a long time [13]. This novel notion has drawn attentions to intracellular Aβ, whose accumulation was recently shown to precede extracellular plaque formation and neuroinflammation and to predict neuronal vulnerability [17]. The fact that Yip7 can retain Aβ intracellularly yet still avoid Aβ toxicity is surprising. It has been proposed that trafficking of Aβ to lysosomes for degradation is a major Aβ clearance pathway, and disturbing this pathway can lead to the accumulation of intracellular Aβ [15, 86]. In both flies and mammals, intracellular Aβ is in turn capable of inducing lysosome rupture, causing neuronal death and extracellular seeding of Aβ [13, 30]. Nevertheless, in our model, Yip7 appears to increase intracellular Aβ accumulation not by impairing the lysosomal function or autophagy (Figure 5A–G), but rather in a more active manner by sequestering Aβ. We speculate that Yip7 may trap Aβ within the lysosomes in a form that is resistant to lysosomal degradation yet does so without triggering increased inflammation, as supported by our behavioral and biochemical analyses and by our transcriptomic data. It is possible that through physical interaction with Aβ, Yip7 restricts the pathogenic aggregation of Aβ by playing a role reminiscent of a molecular chaperone. Indeed, it has been reported that a secreted Hsp70 chaperone can mask Aβ toxicity by promoting the accumulation of non‐toxic aggregates in fly models [69]. Central to building the concept of targeting intracellular amyloid as an alternative therapeutic strategy for AD, future work is required to address the exact subcellular location and aggregation status of Aβ trapped by Yip7, which will enable an understanding of how and why such specific intracellular retention of Aβ by Yip7 does not trigger inflammation in the fly brain.

Serine proteases comprise a large family of endopeptidases with more than 200 members in both flies and humans, whose roles extend far beyond the digestion of dietary contents [40, 87, 88, 89, 90, 91, 92, 93, 94]. In fact, many serine proteases are expressed in the brain and play roles in the development, maintenance, and pathology of the nervous system as previously summarized [33, 95]. Interestingly, earlier work reported the increased production of a‐1‐antichymotrypsin (ACT), a protease inhibitor, in the brains of AD patients. As an amyloid‐associated protein, ACT has a strong stimulatory role in promoting the polymerization of Aβ into amyloid filaments in vitro [96, 97], implying the relevance of serine proteases in Aβ pathogenesis. Of note, the role of proteases in organismal proteostasis and neurodegeneration has only begun to be characterized very recently [31, 32]. Our identification of the serine protease Yip7 in counteracting AD by sequestering intracellular Aβ is completely unexpected in two ways. First, though a well‐defined signal peptide sequence is present at its N‐terminus, experimental evidence shows that Yip7 is not secreted like a typical gut digestive protease but is instead localized to the endosomal/lysosomal compartments in the cytoplasm. Second, instead of degrading intracellular Aβ as would be expected for a serine protease, Yip7 promotes intracellular Aβ sequestration. Further elucidation of the sequence basis that enables protein design to track and trap intracellular Aβ to block its neurotoxicity would be a crucial first step toward targeting intracellular Aβ with a cell biology approach. Our work therefore serendipitously provides an entry point toward this direction, as the PTD of Yip7 appears to be (part of) a novel protein‐targeting signal that marks crucial intermediate stops in the trafficking route of intracellular Aβ. Conceptually, mitigating Aβ neurotoxicity by its intracellular sequestration awaits validation in mammalian AD models, and may inform future designs of anti‐AD strategies.

## 4 Experimental Section

### 4.1 *Drosophila* Stocks

Fruit flies were cultured at 25°C and under 60% humidity with a 12:12 light dark cycle. Conventional fly diet contains per liter 32.3 g yeast, 69.2 g corn flour, 9.2 g soybean meal, 61.5 mL syrup, 1.7 g Nipagin methyl ester, and 7.7 g Agar. Mated females were used throughout the study. Genetic crosses were carried out at 25°C unless otherwise noted and the progenies were collected within three days of eclosion for further analyses. For adult‐onset expression, the TARGET system was used in combination with the indicated Gal4 drivers (*Elav‐Gal4* and *nSyb‐Gal4*) to conditionally express UAS‐linked transgenes [99]. Flies were grown at 21°C to limit Gal4 activity. After 3 days at 21°C, adult flies with the appropriate genotypes were shifted to 29°C, a temperature inactivating the temperature sensitive Gal80's ability to suppress Gal4. Fly strains used in study are as follows. Drivers: *Elav‐Gal4^c155^* (BL458, from Ranhui Duan), *OK107‐Gal4* (BL854, from Hongtao Qin), *nSyb‐Gal4* (BL51944), *GMR‐Gal4* (Kyoto106207). *Mex‐Gal4* (BL91368, from Bruno Lemaitre), *Dilp2‐Gal4* (BL37516). UAS lines: *UAS‐Aβ42^E693G^* (BL33773 and BL33774), *UAS‐Aβ42* (BL33769 and BL64216), *UAS‐yip7‐3xHA* (F003003), *UAS‐Jon65Aiv‐3xHA* (F002953), *UAS‐Jon65Aiii‐3xHA* (F003004), *UAS‐Bace‐3xHA* (F002569), *UAS‐Jon66Cii‐3xHA* (F000772), *UAS‐CG33109‐3xHA* (F002668), *UAS‐CG12374‐3xHA* (F003805), *UAS‐Fit‐HA* [47], *UAS‐hsp70* (human *HSPA1L*) (BL7454 and BL7455), *UAS‐(G_4_C_2_)_36_* (BL58688), *UAS‐poly(GR)_36_‐PO* (BL58692), *UAS‐yip7‐RNAi* (VDRC102226), *UAS‐Jon65Aiii‐RNAi* (VDRC103274), *UAS‐Rab5‐GFP* (BL9775), *UAS‐Rab7‐GFP* (BL42706), *UAS‐KDEL‐GFP* (BL9898), *UAS‐Lamp1‐GFP* (BL42714), *UAS‐ManII‐EGFP* (BL65248), *UAS‐mito‐GFP* (BL8442), *UAS‐pANF‐GFP* [98], *UAS‐syt‐GFP* (BL6925), *UAS‐yip7‐3xHA* (this study), *UAS‐yip7^∆C^‐3xHA* (this study), *UAS‐yip7^∆S^‐3xHA* (this study). Other lines: *w^1118^* (iso31), *VDRC‐KK* control (VDRC60100), *tub‐Gal80^ts^* (BL7018 and BL7019), and *TRE‐dsRed* (BL59011).

### 4.2 Generation of Transgenic Strains

Three transgenic lines expressing Yip7 variants, *UAS‐yip7‐3xHA*, *UAS‐yip7^∆C^‐3xHA*, and *UAS‐yip7^∆S^‐3xHA*, were created in this study. Briefly, the region coding *yip7‐3xHA* was PCR‐amplified from the *UAS‐yip7‐3xHA* transgene (F003003). Based on *yip7‐3xHA*, truncated Yip7 lacking the PTD (*yip7^∆S^‐3xHA*) was also PCR‐amplified; the catalytic triad mutations (His–Asn, Asp–Glu, and Ser–Leu carried in *yip7^∆C^‐3xHA*) were introduced by PCR primers via overlapping PCRs. The three *Yip7‐*HA fragments were then cloned into *pUAST‐attB* vector via homologous recombination (ClonExpress Ultra One Step Cloning Kit, Vazyme). The resultant plasmids were verified by sequencing. Transgenic flies were established by phiC31 integrase‐mediated site‐specific integration. All three transgenes were landed into the *ZH‐86Fb* site via injecting 24749NF line that does not carry the *3xP3‐RFP* marker at UniHuaii (Zhuhai, China), and were established as stable lines using the same genetic crosses so that these transgenes share the same genetic background and are readily comparable. Primer sequences used for cloning are available upon request.

### 4.3 Locomotion Assays

(1) Climbing. Flies were collected and further reared at 29°C for 14 days before being assayed for climbing ability (negative geotaxis). Flies were anesthetized with CO_2_ two days prior to the experiment and divided into groups of 10 flies/tube. For the test, the flies were placed in an empty fly vial (height: 9.5 cm; diameter: 2.4 cm) marked with lengthen scales in 1 cm increment and allowed to acclimate for 30 minutes (min). Then, we gently tapped the flies to the bottom of the tubes and immediately initiated video recording of fly climbing for 8 seconds (s). This was repeated three times with a 5 min interval between trials. From the video, the distance of each fly climbed for 7 s was determined, and the average climbing distance of 10 flies in the same vial was calculated and further averaged from the three technical repeats. At least three biological replicates (the exact number of replicates is indicated in respective figure legends) were conducted for each experiment. The climbing assays were performed at room temperature.

(2) Spontaneous walking. Video tracking was used to assess spontaneous locomotion. Flies were anesthetized on ice and loaded 1 fly/well into a 48‐well plate, with at least 12 flies per genotype. The bottom of the 48‐well plate was filled with 1% agar with 5% sucrose so that flies can only move horizontally. After a 30 min recovery period at room temperature, the flies were placed into the Zebrabox tracking system (ViewPoint) to track fly movement. From the tracking data, we calculated the velocity and total distance travelled by each fly over a 5‐hour (h) period. The tests were routinely done from ZT1‐5 (9 am–13 pm) at room temperature, and the test plates were illuminated during tracking. All experiments were performed in biological triplicate, with the representative tracking data shown in figures.

(3) Activity test with *Drosophila* Activity Monitor. We measured fly activity using DAM2 *Drosophila* Activity Monitor (TriKinetics). 32 female flies per genotype (2–5 days old) were transferred into glass tubes with one end containing a food substrate (5% sucrose and 1% agar) and the other end sealed with a cotton plug. Flies were entrained at 25°C in 12 h: 12 h light/dark (LD) conditions for 2–3 days before experiments. Then, their movements were recorded for three consecutive days by marking their passage through the midpoint of the glass tube detected with infrared beams. Locomotor activity was recorded as raw binary data, where “1” indicates the interruption of the infrared beam and “0” indicates no interruption. Activity counts were collected in 1 min bins and plotted as activity/30 min over ZT times. Data were analyzed using Excel.

### 4.4 Lifespan Assay

Flies were reared at a standard density prior to lifespan experiments. Genetic crosses were performed at 25°C, and progenies were collected and allowed to mate for 3–4 days before being transferred to 29°C to induce Aβ42 deposition. 20–30 flies were placed in each fly vial, and at least 30 flies were used for each genotype. Throughout the course of the experiment, flies were transferred to fresh medium daily, and mortality was recorded daily. Rapamycin feeding was carried out essentially as described [30]. Flies were allowed to mate on normal fly food for 2–3 days and then transferred to fresh food containing 1 µm rapamycin (LC Laboratories) or an equal volume of anhydrous ethanol (vehicle for dissolving rapamycin). Survival curves were generated and analyzed using GraphPad Prism 9 software.

### 4.5 Adult Eye Degeneration

All flies for eye phenotype analysis were crossed and raised at 25°C. Flies were collected and aged for 7 days to measure degenerative eye phenotype by imaging fly eyes under a Nikon SMZ800N Stereo Microscope.

### 4.6 RNA Sequencing and Data Analysis

Total RNA was extracted from heads of 14‐day‐old (for RNA‐seq described in Figure 1) or 10‐day‐old (for RNA‐seq described in Figure 7) flies with genotypes indicated in figures for RNA‐seq analysis. 20 fly heads were used for each sample and biological triplicates were performed. Total RNA extraction was performed using RNAiso Plus (TaKaRa), with all samples processed in parallel to minimize technical variation. RNA purity and quantification were evaluated using the NanoDrop 2000 spectrophotometer (Thermo Scientific). RNA integrity was assessed using the Agilent 2100 Bioanalyzer (Agilent Technologies). Then, the sequencing libraries were constructed using VAHTS Universal V10 RNA‐seq Library Prep Kit (Vazyme) and sequenced on an Illumina Novaseq 6000 platform with 150 bp paired‐end reads. Raw reads of FASTQ format were first processed using fastp and the low‐quality reads were removed to obtain the clean reads that were mapped to the reference genome using HISAT2. FPKM of each gene was calculated and the read counts of each gene were obtained by HTSeq‐count. PCA analysis was performed using R (v3.2.0). Differential expression analysis was performed using the DESeq2. We set *q* value < 0.05 and foldchange > 2 or foldchange < 0.5 as the threshold for significantly differentially expressed gene (DEGs), unless noted otherwise. Hierarchical cluster analysis of DEGs was performed using R (v3.2.0). GO enrichment analysis of DEGs were performed by screening the significantly enriched terms using R (v3.2.0). The transcriptome sequencing was conducted by OE Biotech (Shanghai).

### 4.7 qRT‐PCR Analysis of Gene Expression

Total RNA was extracted from *Drosophila* heads using RNAiso Plus (TaKaRa). cDNA was synthesized using the PrimeScrip RT Reagent Kit (TaKaRa). 100–500 ng total RNA was used for reverse transcription with oligo dT, and the first strand cDNA was diluted 10–20 times with water and further used in real time PCR. Real time PCR was performed in at least duplicates for each sample using SYBR Green (Roche) on a q225 qPCR System from Quantagene (Kubo Technology). Expression values were calculated using the ΔΔCt method and relative expression was normalized to *RpL32*. The expression in control sample was further normalized to 1 in Figure 1I. The primer sequences used are as follows:

*yip7*‐F: 5′‐TCCACCTACGAGGGAAAGA‐3′; *yip7*‐R: 5′‐GGAGTTGGAGATGATGGTAAGG‐3′

*Aβ42*‐F: 5′‐GAATTCCGACATGACTCAGG‐3′; *Aβ42*‐R: 5′‐GCCCACCATGAGTCCAATGA‐3′

*RpL32*‐F: 5′‐TCTGCATGAGCAGGACCTC‐3′; *RpL32*‐R: 5′‐ATCGGTTACGGATCGAACAA‐3′

### 4.8 Western Blots

(1) Standard Western blot analysis. Fly heads were homogenized for 2 h in ice‐cold RIPA buffer containing protease inhibitors, with 35 heads for each sample. Protein samples were analyzed by SDS‐PAGE and transferred to 0.45 µm polyvinylidene difluoride (PVDF) membranes. Immunoblotting was performed using anti‐p62 antibody (1:100; Abcam #ab178440; a gift from Prof. Hongtao Qin). For Aβ detection, 50 heads per group were lysed overnight at 4°C in 100 µL RIPA buffer containing 1% SDS. Protein extracts were dissolved by SDS‐PAGE using Tricine‐SDS‐PAGE Gel Preparation Kit for Small Molecular Weight Proteins (Beyotime #P0531S) and transferred to 0.2 µm PVDF membranes at 70 V for 1 h. To optimize antibody binding, membranes were boiled in 1× TBST for 5 min, then blocked with 5% non‐fat dry milk in TBST for 2 h at room temperature. Primary antibody incubations were performed overnight at 4°C. The following primary antibodies were used: mouse anti‐HA (1:1000, Cell Signaling Technology #3724), mouse anti‐Aβ 6E10 (1:1000; BioLegend #803001), and mouse anti‐Actin (1:1000; Bioss #bsm‐8777 M). Densitometric analysis of blot images was carried out using Fiji and the gray scale of the control sample was normalized to 1.

(2) Western blot analysis for detergent‐insoluble fractions. Western blot analysis for detergent‐soluble and ‐insoluble fractions was performed as previously described [100]. Briefly, the heads of 30 female flies per sample were dissected and homogenized in ice‐cold PBS containing 1% Triton X‐100 and protease inhibitors (Roche #04693132001) for 5 min. The homogenates were centrifuged at 14,000 rcf for 10 min at 4°C, and the supernatants were collected as the Triton X‐100‐soluble fraction. The pellet was further washed twice with 400 µL ice‐cold PBS containing 1% Triton X‐100, followed by centrifugation at 14,000 rcf for 5 min at 4°C. The pellet was then resuspended in RIPA buffer containing 8 m urea and 5% SDS, and centrifuged again at 14,000 rcf for 10 min at 4°C. The resulting supernatants were collected as Triton X‐100‐insoluble fraction and analyzed by SDS‐PAGE using anti‐Ubiquitin (1:1000, Santa Cruz Biotechnology #3936) and anti‐Actin (1:1000, Bioss #bsm‐8777 M) antibodies.

(3) Co‐IP. 60 fly heads were dissected and homogenized in 100 µL lysis buffer (RIPA+1% SDS), except for the Aβ42 + yip7 sample where we used 240 fly heads to facilitate Aβ detection post immunoprecipitation. We used the BeyoMag Anti‐HA Magnetic Beads (Beyotime #P2121) according to the manufacturer's recommendations with minor modifications. The volume of magnetic beads used for the Aβ42 + yip7 sample was proportionally scaled‐up (i.e., 4× of volume used for other samples). Briefly, 10–20 µL HA beads were incubated with 100 µL of homogenate and 400 µL extraction buffer for overnight. After washing, proteins were eluted by boiling in 1× LDS sample buffer (Beyotime #P0731) and detected by Western blot using the rabbit anti‐HA polyclonal antibody and the mouse anti‐Aβ 6E10 monoclonal antibody.

### 4.9 Immunofluorescence

(1) Brain immunostaining. The immunostaining of brains was done as previously described [101]. Fly brains were dissected in ice‐cold PBS and fixed for 2 h in 4% paraformaldehyde in PBS. They were then washed three times in 0.03%Triton X‐100 in PBS at room temperature and blocked for overnight at 4°C in blocking solution (PBS with 2% Triton X‐100 and 10% goat serum), before being incubated in primary antibodies diluted in PBS with 0.25% Triton X‐100 and 1% goat serum for 24 h at 4°C. Samples were washed three times in PBS with 1% Triton X‐100 and 3% NaCl, before being incubated for 24 h with secondary antibodies and DAPI (1:200, Sigma #D9542). For LysoTracker staining, 15 brains per genotype were dissected in PBS, and directly incubated with LysoTracker Red DND‐99 (1:1000; Thermo Fisher Scientific #L7528) in 1× PBS for 5 min. Followed by one PBS wash, the staining was checked under a Zeiss Imager M2 microscope. For TRE‐dsRed intensity, fly brains were dissected in ice‐cold PBS, and fixed for 2 h in 4% paraformaldehyde in PBS. Then, the samples were washed using PBS, mounted in an antifade medium, and imaged on a Zeiss Imager M2 microscope. Quantitative analysis of fluorescence intensity was performed with the ZEN software (Zeiss, Germany). Primary antibodies used were as follows, mouse anti‐Aβ 6E10 (1:200; BioLegend #803001), rabbit anti‐HA (1:10000, Cell Signaling Technology #3724), rabbit anti‐Dcp1 (1:1000; Cell Signaling Technology #9578), rat anti‐Elav (1:1000; DSHB #7E8A10), chicken anti‐GFP (1:1000; Abacm #13970), rabbit anti‐tyrosine hydroxylase (1:1000; Novus Bio #NB300‐109), mouse anti‐NPC/Mab414 (1:500; Abcam #ab24609), and mouse anti‐Lamin (1:500; DSHB #ADL84.12). Secondary antibodies used were goat anti‐mouse Alexa Fluor 488 (1:1000; Invitrogen #A32723), goat anti‐rat Alexa Fluor 555 (1:1000; Invitrogen #A21434), goat anti‐rabbit Alexa Fluor 568 (1:1000; Invitrogen #A11036), and goat anti‐chicken Alexa Fluor 488 (1:1000; Invitrogen #A11039).

(2) Gut immunostaining. Flies were transferred overnight into a classical fly food vial containing a filter paper soaked with a solution consisting of 5% sucrose to clean the digestive tract. Fly guts were dissected in ice‐cold PBS and fixed for at least 1 h at room temperature in 4% paraformaldehyde in PBS. They were washed three times in 0.1%Triton X‐100 in PBS at room temperature, blocked for 2 h at room temperature in blocking solution (0.1%Triton X‐100 and 2% goat serum), then incubated in primary antibody diluted in solution containing 0.1%Triton X‐100 and 2% goat serum overnight at 4°C. After washing for 1 h, samples were incubated with secondary antibodies and DAPI at room temperature for 2 h. After washing three times (15 min each), the guts were mounted in antifade medium and imaged on a Zeiss microscope. Primary antibodies used were rabbit anti‐HA (1:10000; Cell Signaling Technology #3724), chicken anti‐GFP (1:1000; Abacm #13970). Secondary antibodies used were goat anti‐rabbit Alexa Fluor 568 (1:1000; Invitrogen #A11036) and goat anti‐chicken Alexa Fluor 488 (1:1000; Invitrogen #A11039).

### 4.10 Quantification and Statistical Analysis

Quantitative data are shown as mean ± SD from at least three biological replicates of each experiment. For all quantifications, n represents the number of biological replicate and error bar represents SD. In Figures 2B,E,I, 4E, 5B,D, 6C,F,I,H, and Figures S1A and S3C, one dot represents one fly/brain. In Figures 1A,C,H,I and 6A, one dot represents the average distance that ten flies climbed in 7 s. In Figure 4H, one dot was generated from 50 heads. In Figures 2G, 5F, 6E, and Figure S3E, one dot represents 30 heads. In Figures 1J, 4F, and Figure 7B, one dot was generated from 20 heads. Other images shown in figures to characterize gene expression/protein localization are representative of 20–30 individuals and the experiments were repeated at least twice. Statistical significance was determined using either the unpaired *t*‐test for comparison of two groups or one‐way ANOVA with Tukey post hoc tests where multiple comparisons were necessary, with all tests being two‐sided, in GraphPad Prism Software (version 9.5.1), and expressed as *P* values. Survival data were pooled and analyzed in Prism software using the log‐rank test (Figures 2J, 5G, and 6J; Figure S1C,D). Statistical significance is presented as *p < 0.05, **p < 0.01, ***p < 0.001 while ns denotes values whose difference was not significant (*p* > 0.05). Complete details regarding the statistical tests used, sample sizes, and definitions of significance are also reported in the figure legends.

Fluorescent images were minimally processed in Fiji and Adobe photoshop (version CC 2019). Adjustments made to raw images included cropping, annotation, and adjustments to brightness and contrast applied across the entire image. Figures were assembled in Adobe illustrator (version CC 2019). Quantification of Aβ staining intensity in regions of MB (Figure 4D,E; Figure S3B,C), Lamp1‐GFP signal intensity in regions of antennal lobe (AL) (Figure 5C,D) and analysis of relative expression level of *TRE‐dsRed* reporter in the whole brain including optic lobes (Figure 6I; Figure S4A–E) were directly done with the ZEN software. LysoTracker positive speckles were counted manually in the regions of MB (Figure 5A,B). Dying cells (Dcp1^+^ cells) and dopaminergic neurons were manually counted in the entire brain (Figures 2H,I and 6F–H).

## Author Contributions

J. Su and Z. Zhai designed the research, analyzed the data, and wrote the manuscript. P. Wu and Z. Zhai co‐supervised the study. J. Su conducted all the experiments. M. Yang and X. Wang analyzed the RNA‐seq data.

## Conflicts of Interest

The authors declare no conflict of interest.

## Supporting information




**Supporting File**: advs74667‐sup‐0001‐SuppMat.docx.

## Data Availability

All data are included in the article and the Supporting Information. RNA‐seq data have been deposited at SRA and GEO databases under Accession Numbers PRJNA1252987 and GSE300826 and are publicly available. All raw data, details of methods, analytical tools, and scripts are available upon reasonable request. Further information and requests for resources and reagents should be directed to and will be fulfilled by the lead contact, Zongzhao Zhai (zongzhao.zhai@foxmail.com). The data that support the findings of this study are openly available in GEO at https://www.ncbi.nlm.nih.gov/geo/query/acc.cgi?acc=GSE300826, reference number 300826.
