# Supplementary material for: Intracellular Aβ42 Sequestration by a Serine Protease Mitigates Neurotoxicity in a Drosophila Alzheimer's Disease Model
Source: Adv Sci (Weinh). 2026 Mar 18;13(26):e17862. doi: 10.1002/advs.202517862 (PMC13159133; doi:10.1002/advs.202517862)
Supplement: Supplementary file 1 — Supporting File: advs74667‐sup‐0001‐SuppMat.docx. [file ADVS-13-e17862-s001.docx]

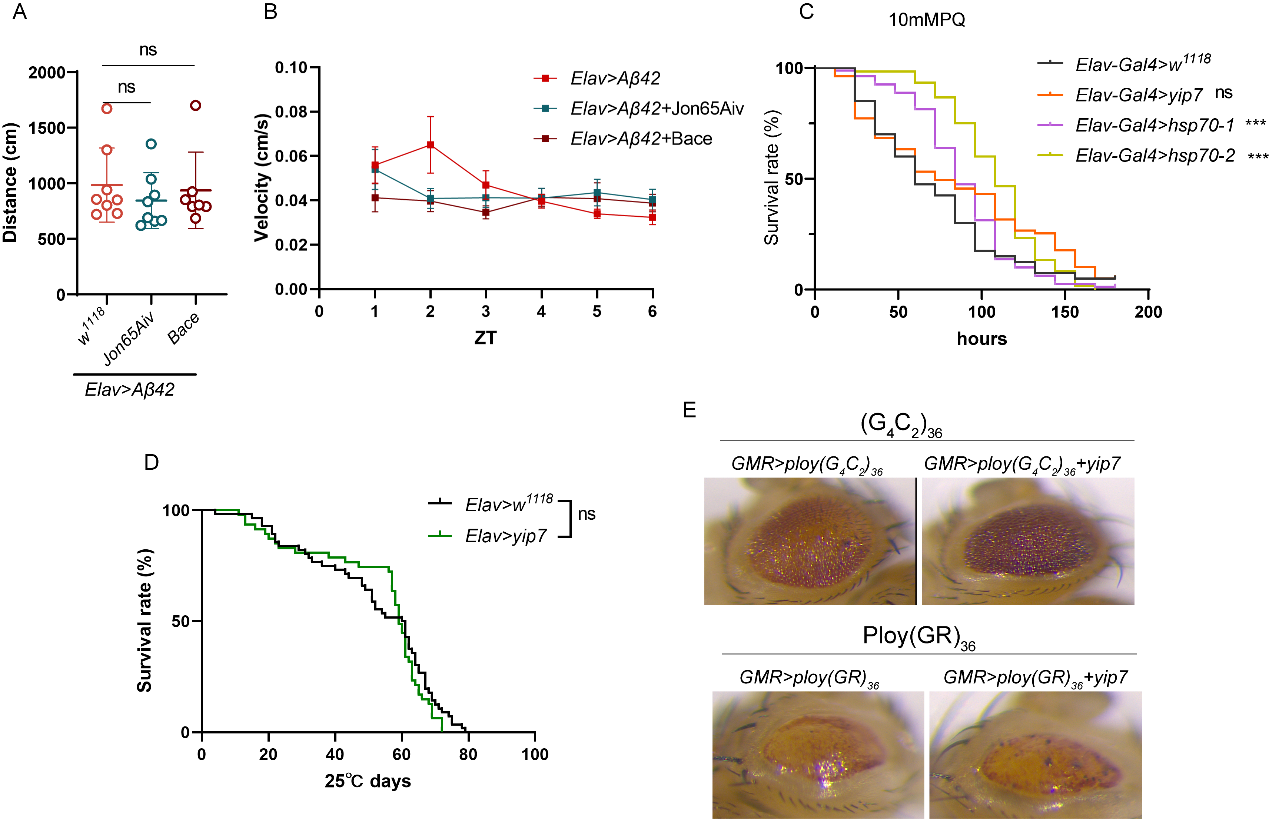


**Figure S1. Yip7 protects against Aβ neurotoxicity but not broader stresses. Related to Figure 2.**

**(A-B)** Spontaneous movements of *Elav>Aβ42* flies and *Elav>Aβ42* flies co-expressing Jon65Aiv and Bace monitored with the ViewPoint Zebrabox tracking system for 5 hours. Shown are the quantification of the distance travelled (A) and velocity (B) over ZT times. Each dot represents one fly in A; one-way ANOVA with Tukey’s *post hoc* test. (**C**) Survival analysis of flies with the indicated genotypes upon ingesting10mM paraquat (PQ). *n* > 40 flies for each genotype. (**D**) Lifespan analysis of control flies and flies pan-neuronally expressing Yip7 using *Elav-Gal4* at 25°C. *n* = 60 flies for each genotype; Log-rank (Mantel-Cox) test. (**E**) Representative images showing eye toxicity due to expressing *poly(G_4_C_2_)_36_* (left) and *poly(GR)_36_* (right) using *GMR-Gal4.* Note that expressing (*G_4_C_2_*)*_36_* makes both *G_4_C_2_* RNA repeats and dipeptide repeats, while expressing *poly(GR)_36_* only makes protein repeats. In both cases, co-expressing Yip7 did not suppressed eye degeneration. Data are presented as mean ± SD in B. ****P* < 0.001; ns, not significant.


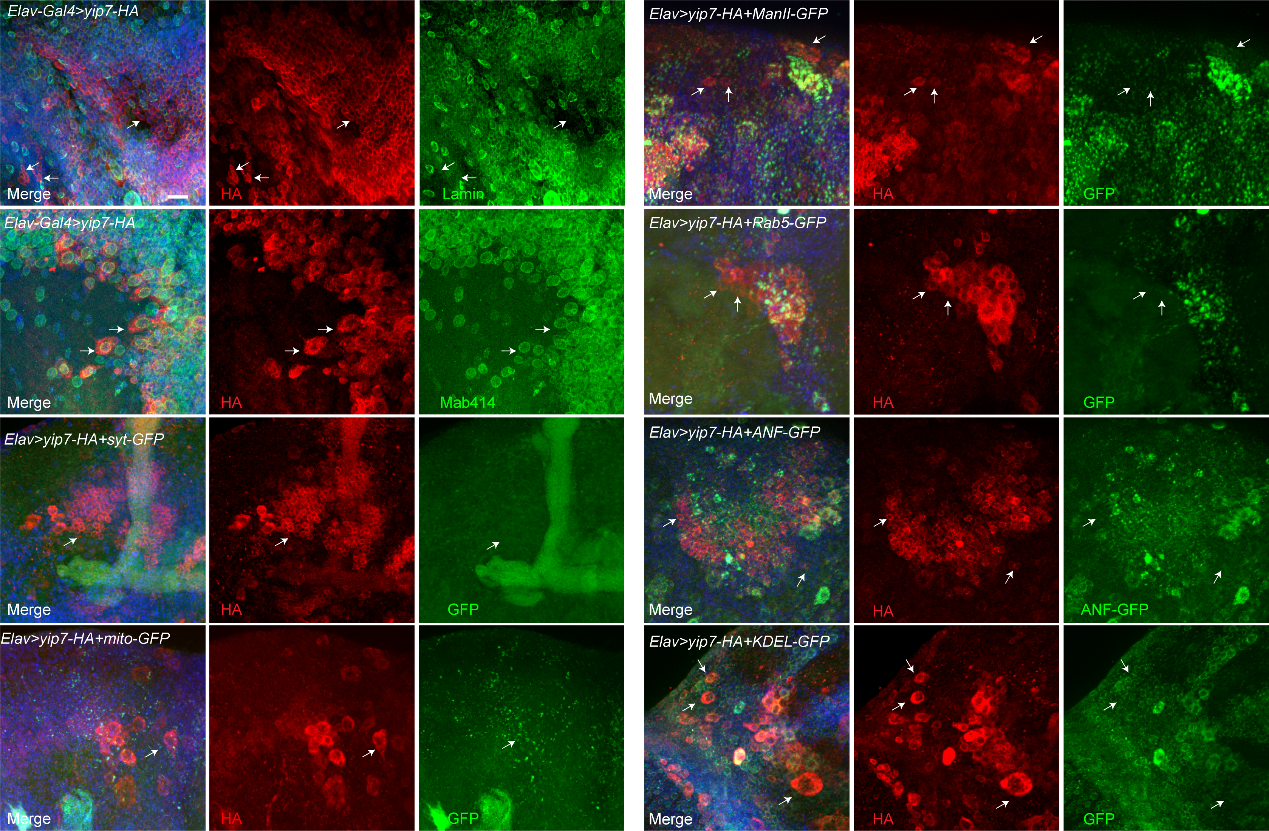


**Figure S2. Subcellular Localization of Yip7. Related to Figure 3.**

Co-localization of Yip7-HA (HA staining) with specific markers for subcellular compartments. Lamin and Mab414 are antibody staining, and others are GFP staining of *UAS-marker-GFP* expressed with *Elav-Gal4*. Lamin and Mab414: nuclear membrane marker; syt-GFP: synaptic vesicle marker; mito-GFP: mitochondrial marker; ManII-GFP: Golgi complex marker; Rab5-GFP: early endosome marker; ANF-GFP: dense vesicle marker; KDEL-GFP: endoplasmic reticulum marker. Arrows indicate locations with the presence of Yip7 but absence of respective markers. Scale bar 26μm.


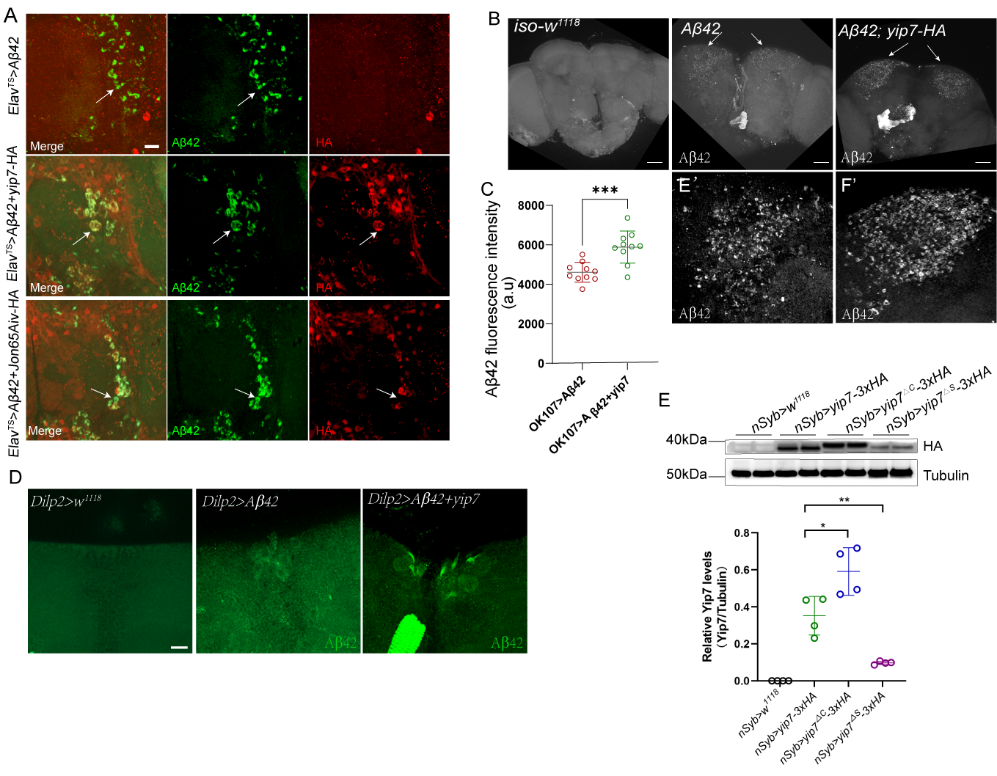


**Figure S3. Yip7 promotes neuronal sequestration of Aβ. Related to Figure 4.**

(**A**) Co-localization of Yip7-HA and another serine protease Jon65Aiv-HA with Aβ42 expressed pan-neuronally with *Elav-Gal4*. Note the complete overlap of Yip7-HA with Aβ42 expression but the limited overlap of Jon65Aiv-HA with Aβ42. (**B**) Representative images of Aβ42 staining in control flies, flies expressing *Aβ42* alone or simultaneously co-expressing *yip7* in mushroom bodies using *OK107-Gal4* for 21 days at 29°C. (**C**) Quantification of Aβ42 staining intensity in mushroom bodies. Each dot indicates one brain. (**D**) Yip7 expression also led to Aβ42 retention in insulin-producing cells (IPCs) using *Dilp2-Gal4* as a driver. (**E**) A representative blot of four independent replicates and quantification of protein levels in heads of flies pan-neuronally expressing each of the three Yip7-3xHA variants using *nSyb-Gal4*. Statistical analyses were conducted using Student’s *t*-test in C and using one-way ANOVA with Tukey’s *post hoc* test in E. All data are presented as mean ± SD. ****P* < 0.001; ***P* < 0.01; **P* < 0.05; ns, not significant. Scale bars 26μm (A), 50μm (B) and 23μm (D).


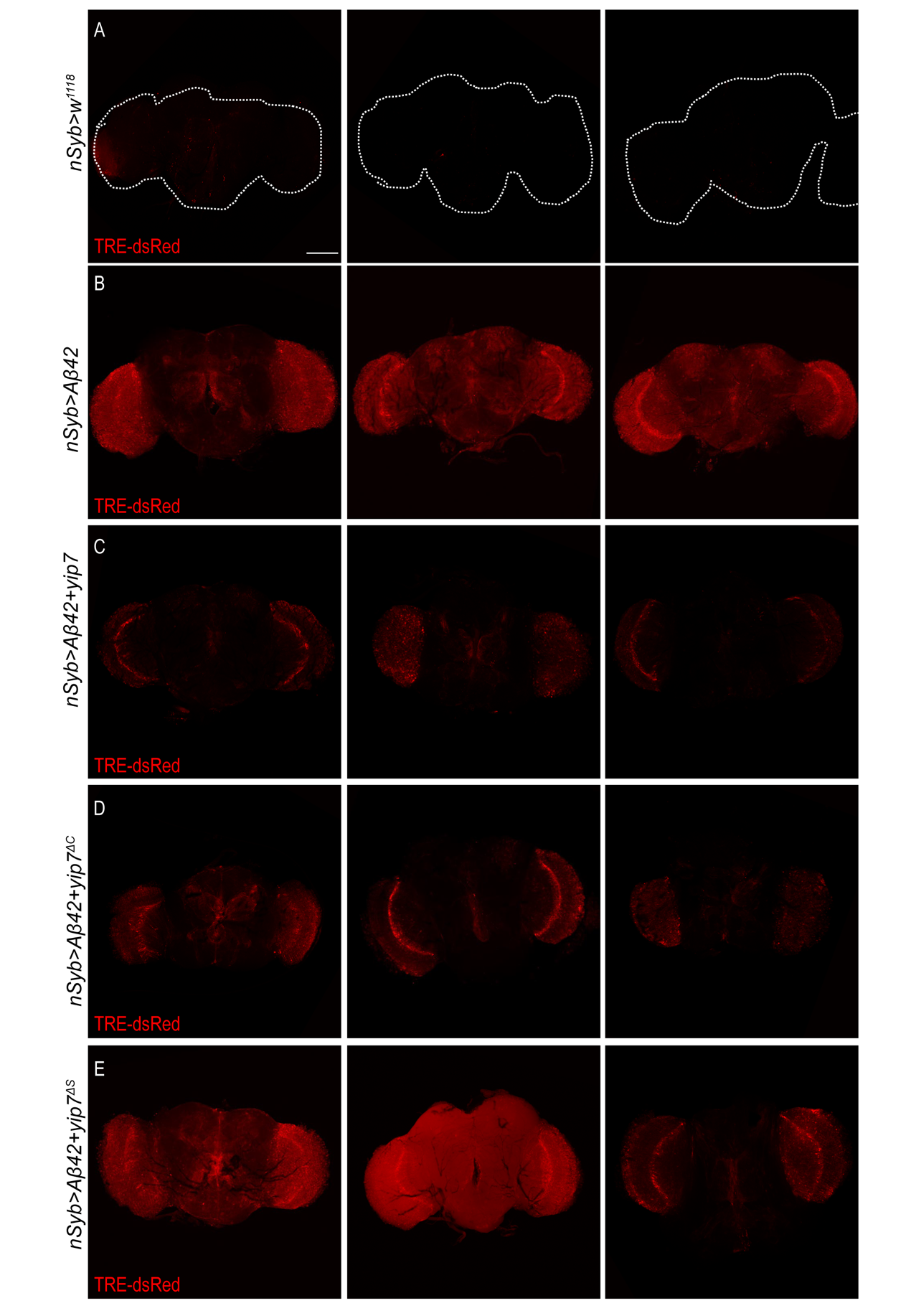


**Figure S4. Brain senescence marker *TRE-dsRed* is suppressed by Yip7. Related to Figure 6.**

(**A-E**) Live fluorescence of *TRE-dsRed* reporter in the brain of flies expressing *Aβ42* alone or simultaneously co-expressing *yip7*, *yip7^ΔC^*, or *yip7^ΔS^* pan-neuronally using *nSyb-Gal4* for 10 days at 29°C. All images were taken at the same microscope settings. Three representative brains were shown for each genotype. Scale bar 50μm.


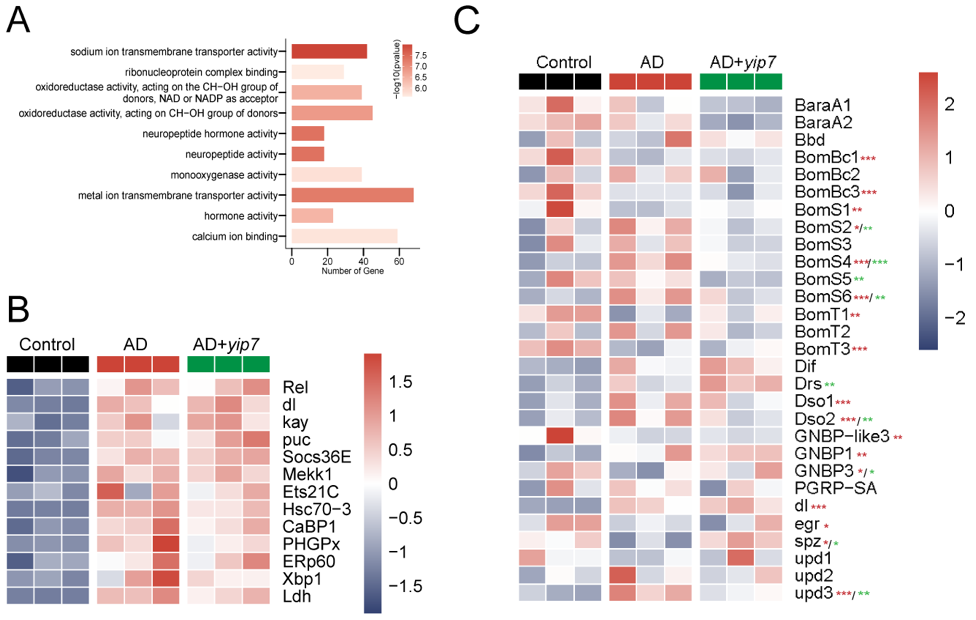


**Figure S5. Intracellular retention of Aβ by Yip7 does not increase the expression of inflammation-associated genes. Related to Figure 7.**

(**A**) GO terms (molecular function, MF) enriched for genes whose expression is significantly changed in AD vs Control but not further altered by co-expressing Yip7. (**B**) Heatmap of expression profiles of known markers of tissue inflammation and stress response. (**C**) Heatmap of expression profiles of genes related to *Drosophila* Toll signaling and of genes showing cytokine-like features (*egr*, *spz* and *upd1-3*). Differentially expressed genes (DEGs) in AD vs Control comparison are indicated with red stars, while DEGs in AD+Yip7 vs AD comparison are indicated with green stars. ****P* < 0.001; ***P* < 0.01; ***P* < 0.05.
